# Protective Effects of Naringin–Dextrin Nanoformula against Chemically Induced Hepatocellular Carcinoma in Wistar Rats: Roles of Oxidative Stress, Inflammation, Cell Apoptosis, and Proliferation

**DOI:** 10.3390/ph15121558

**Published:** 2022-12-14

**Authors:** Eman E. Mohamed, Osama M. Ahmed, Adel Abdel-Moneim, Khairy M. A. Zoheir, Basem H. Elesawy, Ahmad Al Askary, Ahmed Hassaballa, Ahmed A. G. El-Shahawy

**Affiliations:** 1 Physiology Division, Faculty of Science, Beni-Suef University, P.O. Box 62521, Beni-Suef 2722165, Egypt; 2 Cell Biology Department, Biotechnology Research Institute, National Research Centre, Cairo 12622, Egypt; 3 Department of Pathology, College of Medicine, Taif University, P.O. Box 11099, Taif 21944, Saudi Arabia; 4 Department of Clinical Laboratory Sciences, College of Applied Medical Sciences, Taif University, P.O. Box 11099, Taif 21944, Saudi Arabia; 5 Nutrition and Food Science, College of Liberal Arts and Sciences, Wayne State University, Detroit, MI 48202, USA; 6 ZeroHarm L.C., Farmington Hills, Farmington, MI 48333, USA; 7 Materials Science and Nanotechnology Department, Faculty of Postgraduate Studies for Advanced Sciences (PSAS), Beni-Suef University, Beni-Suef 2722165, Egypt

**Keywords:** diethylnitrosamine, acetylaminofluorene, naringin, naringin–dextrin nanoparticles, anti-inflammatory effects, antiapoptotic effects

## Abstract

Nanotechnology holds great promise for the development of treatments for deadly human diseases, such as hepatocellular carcinoma (HCC). In the current study, we compared the hepatoprotective effects of naringin–dextrin nanoparticles (NDNPs) against HCC in male Wistar rats with those of pure naringin and investigated the underlying cellular and molecular mechanisms. HCC was induced by intraperitoneal injection of diethylnitrosamine (DEN, 150 mg/kg body weight (b.w.) per week) for two weeks, followed by oral administration of 2-acetylaminofluorene (2AAF, 20 mg/kg b.w.) four times per week for three weeks. DEN/2AAF-administered rats were divided into three groups that respectively received 1% carboxymethyl cellulose (as vehicle), 10 mg/kg b.w. naringin, or 10 mg/kg b.w. NDNP every other day by oral gavage for 24 weeks. Both naringin and NDNP significantly attenuated the harmful effects of DEN on liver function. Both compounds also suppressed tumorigenesis as indicated by the reduced serum concentrations of liver tumor markers, and this antitumor effect was confirmed by histopathological evaluation. Additionally, naringin and NDNP prevented DEN-induced changes in hepatic oxidative stress and antioxidant activities. In addition, naringin and NDNP suppressed inflammation induced by DEN. Moreover, naringin and NDNP significantly reduced the hepatic expression of Bcl-2 and increased Bax, p53, and PDCD5 expressions. Naringin and NDNP also reduced expression of IQGAP1, IQGAP3, Ras signaling, and Ki-67 while increasing expression of IQGAP2. Notably, NDNP more effectively mitigated oxidative stress and inflammatory signaling than free naringin and demonstrated improved antitumor efficacy, suggesting that this nanoformulation improves bioavailability within nascent tumor sites.

## 1. Introduction

Hepatocellular carcinoma (HCC) is the most common form of liver cancer and among the most frequent causes of cancer-related death worldwide [1]. The development of HCC involves both hyperproliferation of hepatocytes and concomitant angiogenesis [2,3]. These changes in cellular behavior can be induced by alcoholic cirrhosis; nonalcoholic fatty liver disease; intake of mycotoxin-contaminated grains; exposure to various air, food, and water-borne nitrosamines; and infection by hepatitis virus A, B, and C [4,5].

Diethylnitrosamine (DEN) is a chemical compound found in cigarette smoke, cheese, water, cured and fried foods, chemicals used in the farming industry, pharmaceutical products, and cosmetics that is known to cause cancers of the lung, esophagus, and liver in experimental animals [6,7]. In addition, DEN damages liver tissue [8] by inducing the generation of reactive oxygen species (ROS), thereby causing oxidative stress and triggering inflammation [9]. It is well-known that malignant cells can maintain higher intracellular ROS levels than normal cells [10,11]. Overproduction of ROS in mitochondria can damage organelles, including mitochondria, which in turn activates cell death and inflammatory pathways [12]. Moreover, ROS-induced damage of DNA results in the accumulation of genetic and epigenetic changes and shifts the balance between signaling pathways that suppress and those that promote carcinogenesis [13]. There are also strong links between oncogenic and proinflammatory signaling pathways, and many oncogenic chemicals are known to activate genes controlling inflammatory cell signaling pathways, as well as processes involved in carcinogenesis [14,15]. Thus, the various infectious agents, metabolic disorders, and chemical compounds believed to cause HCC may initiate tumorigenesis via induction of oxidative damage and inflammation.

Apoptosis is mediated by intrinsic and extrinsic signaling pathways that can be activated by numerous factors, including cellular stress and DNA damage [16]. The biological processes of differentiation, pathogen defense, and tissue homeostasis all involve apoptosis. Dysregulation of apoptosis causes many diseases, including cancer [17]. Apoptosis is controlled by the equilibrium of prosurvival and proapoptotic proteins. Cancer is characterized by uncontrolled growth of the cell in an abnormal manner. In addition to uncontrolled cell proliferation, evasion of apoptosis is one of the hallmarks of cancers that promote tumor formation and progression. One form of anticancer therapeutic, BH3-mimetic drugs, has been developed to directly activate the apoptosis machinery in malignant cells [18]. Moreover, selection of cancer cells resistant to apoptosis induced by current chemotherapies can lead to recurrence, treatment failure, metastasis, and, ultimately, cancer-related death.

There is no broadly effective chemotherapy or surgical intervention for HCC [19], so there is an increasing emphasis on potential preventative strategies, including the use of natural products and herbal medicines [20]. Naringin is a flavanone glycoside isolated from citrus fruit with numerous beneficial bioactivities, including antioxidant, antiinflammatory, anticancer, antimicrobial, and antiviral effects [21]. A major limitation of most traditional oral and injectable chemotherapeutic drugs is that they are non-selective in both tumor and nontumor tissues when exposed to similar concentrations, which reduces the tolerable dose. The use of nano-biotechnology for the design of formulations with enhanced tumor targeting, termed nano-oncology [22,23], may help circumvent this limitation. Natural product-based nanoformulations, in particular, may have even better efficacy and fewer side effects than synthetic nanoformulations [24].

Indeed, the therapeutic effects of chemotherapeutic drugs can be improved and nontarget toxicity reduced using nanocarriers [25,26]. A new naringin–dextrin nanoparticle (NDNP) formulation was recently described with improved antiinflammatory and anticancer effects against human HCC cell lines compared to free naringin [27]. The current study was designed to develop and test a new naringin–dextrin nanoparticle (NDNP) formulation with improved anticancer efficacy against DEN/2AAF (2-acetylaminofluorene)-induced hepatocarcinogenesis in male Wistar rats. We demonstrate superior efficacy compared to free naringin due to enhanced antioxidant, antiinflammatory, antiproliferative, and proapoptotic activities.

## 2. Results

### 2.1. Characterization, Entrapment Efficiency (EE), and Release Profile

The data concerning the X-ray diffraction (XRD), Fourier-transformation infrared (FTIR) spectra analysis, surface morphology by transmission electron microscopy (TEM), Zetasizer and zeta potential measurements, EE, and release profile were described in detail in our previous publication [27]. The figures representing these data are included in the Supplementary Materials.

### 2.2. Effect of Free Naringin and NDNP on DEN/2AAF-Induced Liver Damage

Rats treated with DEN/2AAF alone exhibited significantly (*p* < 0.05) elevated serum alanine transaminase (ALT), aspartate transaminase (AST), and alkaline phosphatase (ALP) activities and total bilirubin concentrations, as well as significantly reduced serum albumin, compared to untreated normal rats, changes indicative of liver tissue damage. However, treatment with naringin or NDNP following DEN/2AAF significantly reduced serum ALT, AST, ALP, and total bilirubin and significantly increased serum albumin compared to DEN/2AAF treatment alone (Table 1), suggesting marked protection against hepatotoxicity.

**Figure 1:**
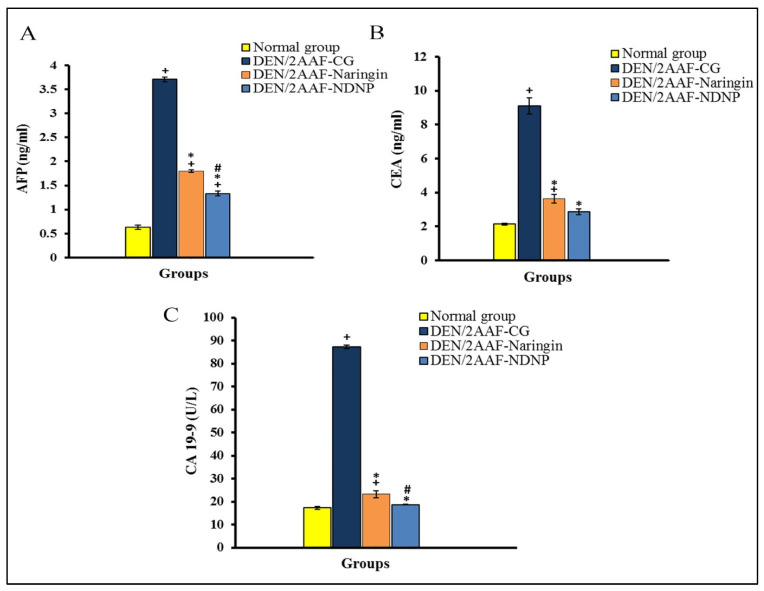
Naringin and naringin–dextrin nanoparticles (NDNPs) reduced serum tumor biomarker concentrations in DENA/2AAF-treated rats. (**A**) Serum AFP. (**B**) Serum CEA. (**C**) Serum CA19.9. Data are expressed as means ± standard error. Number of detected samples in each group was six. ^+^ Significant compared to normal group; * significant compared to DEN/2AAF-CG; and ^#^ significant compared to DEN/2AAF + naringin group at *p* < 0.05. AFP: alpha fetoprotein; CEA: carcinoembryonic antigen; CA19.9: carbohydrate antigen 19.9.

Serum concentrations of the tumor markers alpha fetoprotein (AFP), carcinoembryonic antigen (CEA), and carbohydrate antigen 19.9 (CA19.9) were also significantly elevated in DEN/2AAF-treated rats compared to untreated normal rats, and these responses were also significantly reversed by naringin or NDNP (Figure 1).

### 2.3. Effect of Free Naringin and NDNP on DEN/2AAF-Induced Changes in Hepatic Oxidative Stress and Antioxidant Activities

**Figure 2:**
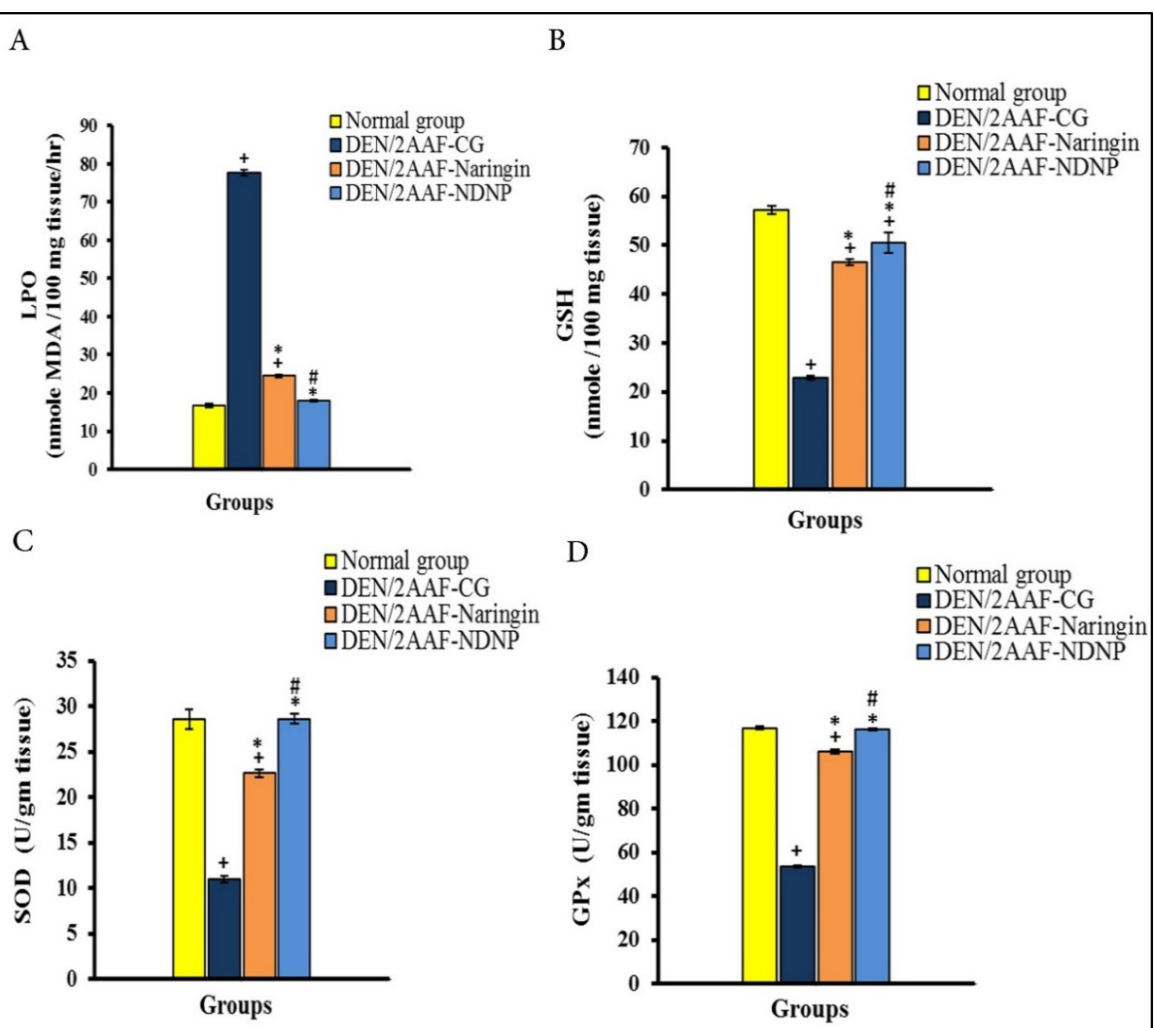
Naringin and NDNP suppressed hepatic oxidative stress in HCC model rats. (**A**) LPO. (**B**) GSH concentration. (**C**) SOD activity. (**D**) GPx activity. Data are expressed as means ± standard error. Number of detected samples in each group was six. ^+^ Significant compared to normal group; * significant compared to DEN/2AAF-CG; and ^#^ significant compared to DEN/2AAF + naringin group at *p* < 0.05. LPO: lipid peroxidation; GSH: reduced glutathione; SOD: superoxide dismutase; GPx: glutathione peroxidase.

Administration of DEN/2AAF significantly elevated lipid peroxidation (LPO) and reduced glutathione (GSH) content in liver tissue compared to normal rats, while the treatment with naringin and NDNP produced a significant decrease in LPO and a significant increase in GSH content compared to the DEN control group. Moreover, NDNP was more effective than naringin in reducing elevated liver LPO and increasing GSH content (Figure 2A,B).

Administration of DEN/2AAF also significantly reduced superoxide dismutase (SOD) and glutathione peroxidase (GPx) activities compared to normal rats. Further, treatment with naringin and NDNP restored SOD and GPx activities (Figure 2C,D). Collectively, these results indicate that NDNP and naringin can mitigate DEN/2AAF-induced oxidative stress by maintaining cellular antioxidant capacity and that NDNP is more efficacious than free naringin.

### 2.4. Effect of Free Naringin and NDNP on DEN/2AAF-Induced Changes in Hepatic Tumor Necrosis Factor Alpha (TNF-α), Interleukin 1 Beta (IL-1β), and Nuclear Factor E2-Related Factor 2 (NRF2) Levels

Rats treated with DEN/2AAF also exhibited a significant increase in liver tissue concentrations of proinflammatory cytokines TNF-α and IL-1β, as well as a reduction in NRF2 level, a transcription factor essential for orchestrating cellular stress responses, compared to normal rats. Subsequent naringin or NDNP treatment reversed both the increases in TNF-α and IL-1β, as well as the reduction in NRF2, compared to DEN/2AAF-administration alone. Treatment with NDNP was more effective than treatment with free naringin (Table 2), suggesting greater efficacy in reducing DEN/2AAF-induced inflammation.

### 2.5. Effect of Free Naringin and NDNP on DEN/2AAF-Induced Changes in Hepatic Nuclear Factor-Kappa B (NF-κB) and Interleukin-8 (IL-8) Expressions

**Figure 3:**
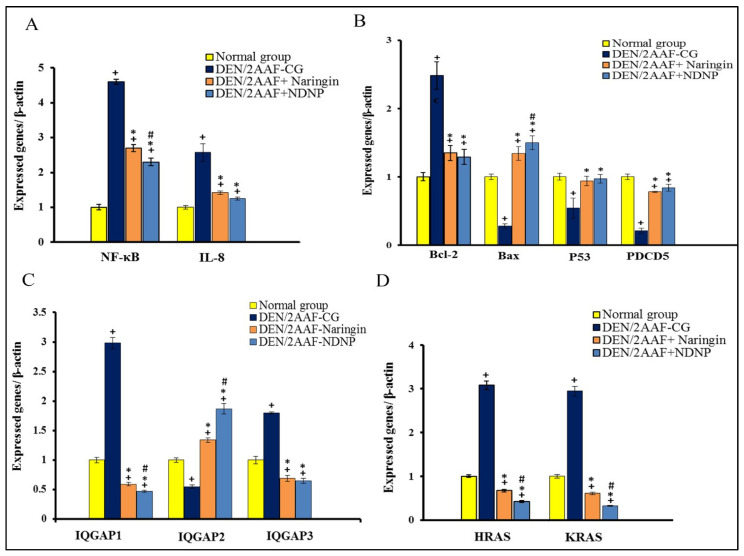
Effects of naringin and NDNP on (**A**) NF-κB and IL-8 mRNA expression; (**B**) antiapoptotic Bcl-2 expression and proapoptotic Bax, p53, and PDCD5 expression; (**C**) IQGAP1, IQGAP2, and IQGAP3 mRNA expression; and (**D**) HRAS and KRAS mRNA expression in DENA/2AAF-treated rats. Data are expressed as means ± standard error. Number of detected samples in each group was six. ^+^ Significant compared to normal group; * significant compared to DEN/2AAF-CG; and ^#^ significant compared to DEN/2AAF + naringin group at *p* < 0.05. NF-κB: nuclear factor-kappa B; IL-8: interleukin-8; Bcl-2: B-cell lymphoma-2; Bax: Bcl-2-associated X protein; p53: tumor suppressor protein 53; PDCD5: programmed cell death 5; IQGAP1: isoleucine–glutamine motif-containing GTPase-activating protein 1; IQGAP2: isoleucine–glutamine motif-containing GTPase-activating protein 2; IQGAP3: Isoleucine–glutamine motif-containing GTPase-activating protein 3; HRAS: Harvey rat sarcoma viral oncogene homolog; KRAS: Kirsten rat sarcoma viral oncogene homolog.

Administration of DEN/2AAF alone also induced a significant elevation in hepatic NF-κB and IL-8 expressions compared to normal rats, and both responses were reversed by subsequent naringin or NDNP treatment (Figure 3A).

### 2.6. Effects of Free Naringin and NDNP on mRNA Expression of Antiapoptotic and Proapoptotic Biomarkers

Administration of DEN/2AAF alone significantly upregulated expression of the antiapoptotic gene Bcl-2 compared to untreated normal rats, a response reversed by free naringin and NDNP. Conversely, DEN/2AAF downregulated the expressions of proapoptotic Bcl-2-associated X protein (Bax), tumor suppressor protein 53 (p53), and programmed cell death 5 (PDCD5) expressions compared to normal rats, and again these changes were significantly reversed by free naringin or NDNP (Figure 3B).

### 2.7. Effects of Free Naringin and NDNP on DEN/2AAF-Induced Changes in Hepatic Isoleucine–Glutamine Motif-Containing GTPase-Activating Protein 1, 2 and 3 (IQGAP1, IQGAP2, and IQGAP3) Expressions

Rats treated with DEN/2AAF alone showed elevated expression of IQGAP1 and IQGAP3 and reduced expression of IQGAP2. Again, these changes were reversed by naringin and NDNP, with NDNP demonstrating greater efficacy (Figure 3C).

### 2.8. Effects of Free Naringin and NDNP on DEN/2AAF-Induced Changes in Hepatic Harvey Rat Sarcoma Viral Oncogene Homolog (HRAS) and Kirsten Rat Sarcoma Viral Oncogene Homolog (KRAS) Expressions

Both HRAS and KRAS mRNA expressions were significantly upregulated by DEN/2AAF, and both responses were significantly reversed by subsequent free naringin or NDNP treatment (Figure 3D).

### 2.9. Effects of Free Naringin and NDNP on DEN/2AAF-Induced Changes in Hepatic Proliferator Protein (Ki-67) Expression

**Figure 4:**
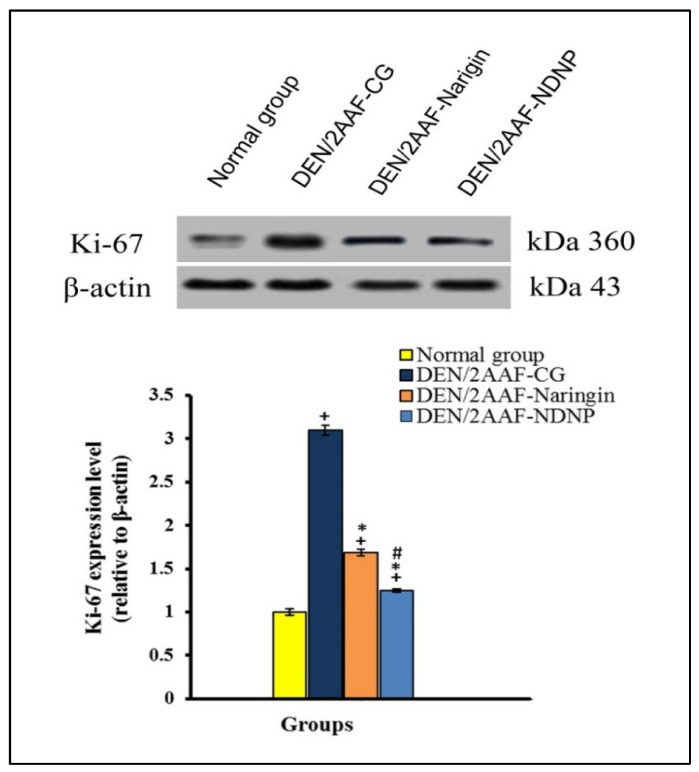
Effect of naringin and NDNP on liver Ki-67 expression in DENA/2AAF-treated rats. Data are expressed as means ± standard error. Number of detected samples in each group was 3. ^+^ Significant compared to normal group; * significant compared to DEN/2AAF-CG; and ^#^ significant compared to DEN/2AAF + naringin group at *p* < 0.05. Ki-67: Proliferator protein.

The DEN/2AAF treatment group also exhibited hepatic overexpression of Ki-67 (*p* < 0.05), suggesting cellular hyperproliferation and being consistent with induction of tumor markers, while both free naringin and NDNP treatments reversed this increase (*p* < 0.05) (Figure 4).

### 2.10. Improvements in DEN/2AAF-Induced Liver Histopathology by Free Naringin and NDNP

**Figure 5:**
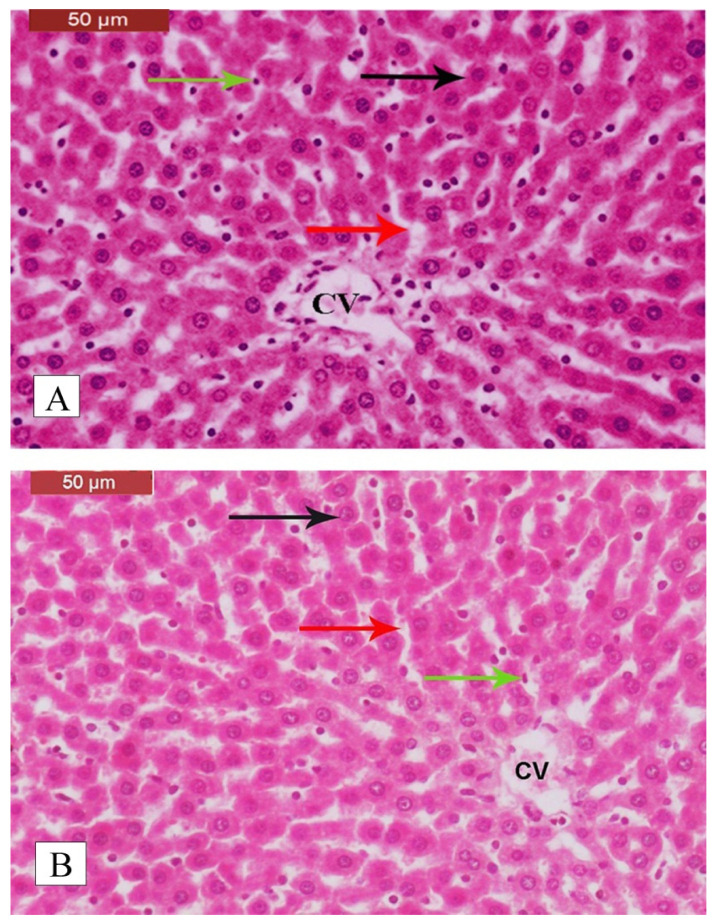
Histological features of liver sections from untreated control rats. (**A**,**B**) Sections stained with H&E showing intact lobules with portal triads among them. Each lobule consisted of cords formed from regularly arranged hepatocytes (black arrow) enclosing sinusoids (red arrow) lined with Kupffer cells (green arrow) and a central vein (CV) located in the center (H&E stain, scale bar: 50 µm).

**Figure 6:**
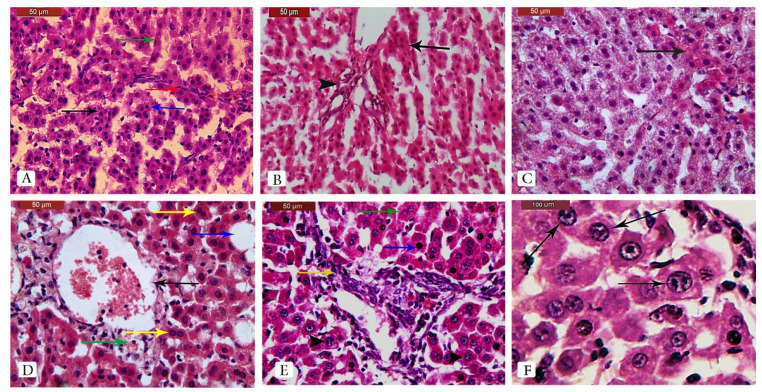
Liver sections from DEN/2AAF-adminstered rats showing extensive histopathology, including disruption of the lobular structure, degeneration, and tumorigenesis. (**A**) Disruption of hepatic lobule structure. Tumor cells appeared well-differentiated or resembled hepatocytes and formed trabeculae (arrow), cords (green arrow), and nests (blue arrow). Note the growing septa between hepatic lobules (red arrow) (H&E stain, scale bar: 50 µm). (**B**) Disruption of the normal structure of the hepatic lobule (arrowhead) and tumor cells arranged in glandular or lamellar form (arrow) (H&E stain, scale bar: 50 µm). (**C**) Clear hepatocyte foci, deeply eosinophilic hepatocyte foci, vacuolated hepatocytes, and dysplastic hepatocytes with mitotic figures (H&E stain, scale bar: 50 µm). (**D**) Dilated central veins (arrow), some with signet ring appearance (green arrow), binucleated hepatocytes (yellow arrow), and hepatocytic steatosis (blue arrow) (H&E stain, scale bar: 50 µm). (**E**) Fibrotic tissue with inflammatory cells (yellow arrow), hepatocytes with dark shrunken nuclei (blue arrow), activated Kupffer cells, binucleated hepatocytes (green arrow), and hepatocytes with large hyperchromatic nuclei and prominent enlarged nucleoli (arrowhead) (H&E stain, scale bar: 50 µm). (**F**) Large hyperchromatic nuclei with prominent multiple nucleoli (arrow) (H&E stain, scale bar: 100 µm).

Examination of H&E-stained liver sections from control rats revealed normal histological features, including typical lobules with portal triads among them. Each lobule consisted of cords formed of regularly arranged hepatocytes enclosing the sinusoids and surrounded by Kupffer cells, with a central vein located in the center of the lobule (Figure 5A,B). In contrast, liver sections of DEN/2AAF-adminstered rats showed a disrupted hepatic lobular structure. Further, well-differentiated tumor cells resembling hepatocytes were observed forming trabeculae, cords, and nests. There were also growing septa between the hepatic lobules (Figure 6A). In regions with disruption of the normal hepatic lobular structure, the tumor cells formed glandular or lamellar structures (Figure 6B). Clear hepatocyte foci, vacuolated hepatocytes, deeply eosinophilic hepatocyte foci, and dysplastic hepatocytes with mitotic figures were also found (Figure 6C), as well as dilated central veins (some with a signet ring appearance), binucleated hepatocytes, and hepatocytic steatosis (Figure 6D). There were also increases in fibrous tissue foci containing inflammatory cells, hepatocytes with dark shrunken nuclei, activated Kupffer cells, binucleated hepatocytes, and hepatocytes with large hyperchromatic nuclei and a prominent enlarged nucleolus (Figure 6E) or multiple nucleoli (Figure 6F).

**Figure 7:**
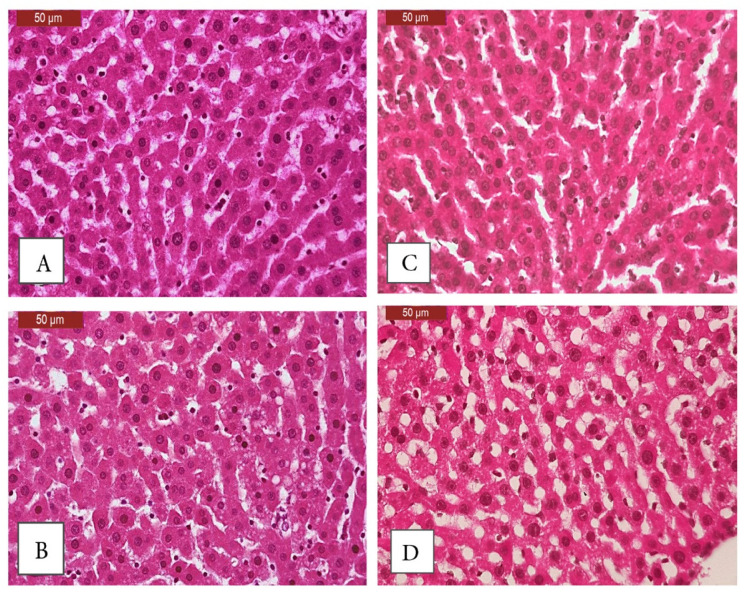
(**A**,**B**) Liver sections from DEN/2AAF-administered rats treated with naringin showing marked amelioration of histopathology and fewer degenerated and vacuolated hepatocytes, together with less extensive dysplasia, milder hepatic sinusoid dilation, fewer proliferating Kupffer cells, and maintenance of normal lobular architecture. Some sporadic tumor cells were found but rarely in a glandular form (H&E stain, scale bar: 50 µm). (**C**,**D**) NDNP treatment showing similar hepatoprotection as free naringin but with an absence of tumor cells (H&E stain, scale bar: 50 µm).

The liver sections of DEN/2AAF-administered rats treated with naringin showed marked amelioration of liver degeneration—with fewer vacuolated hepatocytes, reduced dysplasia, dilatation of hepatic sinusoids, pyknosis of hepatic cells, and proliferation of Kupffer cells—and preserved lobular architecture. Tumor cells were sporadic and rarely in a glandular form (Figure 7A,B). Similarly, the liver sections of DEN/2AAF-administered rats treated with NDNP also showed reduced degeneration, hepatocyte vacuolization, dysplasia, and congestion of hepatic sinusoids, as well as preserved lobular architecture. In addition, however, there was an absence of tumor cells (Figure 7C,D). Histopathological scores of liver lesions in the normal rats, the DEN control group, and the rats treated with naringin and NDNP are shown in Table 3.

## 3. Materials and Methods

### 3.1. Chemicals

Naringin, DEN, 2AAF, and dextrin were obtained from Sigma Chemicals (St. Louis, MO, USA) and stored at 2–4 °C. All other reagents used were of analytical grade.

### 3.2. Preparation and Characterization of Dextrin–Naringin Nanoparticles

We prepared naringin-loaded dextrin using the technique described by Manchu et al. [28]. Briefly, an aqueous nanoemulsion of dextrin and naringin was produced at a final concentration of 5% (*w*/*w* or 0.2 mg/mL) through 1 min of continuous ultrasonication. The crosslinking agent formaldehyde was then added at precise dextrin molar ratios, ultrasonically homogenized for 30 min, and stirred for 12 h. The crosslinked dextrin–naringin nanoemulsion was then precipitated with ethanol 99% (*v*/*v*), followed by 45 min of centrifugation at 4 °C and 14,000 rpm. The nanoemulsion was then freeze-dried for 48 h and stored as a powder at 4 °C. The methods used for characterization of NDNP were described in our previous publication [27]. These methods include XRD, FTIR spectroscopy, TEM, Zetasizers and zeta potential measurements, and ultraviolet–visible (UV–Vis) spectrometery. In addition, EE and the release of naringin from dextrin were also investigated in this study.

### 3.3. Experimental Animals

Adult male Wistar rats weighing 100–120 g were acquired from the Animal Housing Facility of the Egyptian Organization for Biological Products and Vaccines (VACSERA, Helwan, Cairo, Egypt). They were maintained under observation for two weeks before experiments to ensure that any infections were eradicated and then housed at the Zoology Department Animal House at the Faculty of Science, Beni-Suef University, Egypt, under normal room temperature (20–25 °C) and a daily lighting cycle (10–12 h/day) with ad libitum access to water and food. Animal care and experimental protocols conformed to the regulations of the Faculty of Science Experimental Animal Ethics Committee (ethical approval number: 020-91).

### 3.4. Experimental Design and Animal Grouping

**Figure 8:**
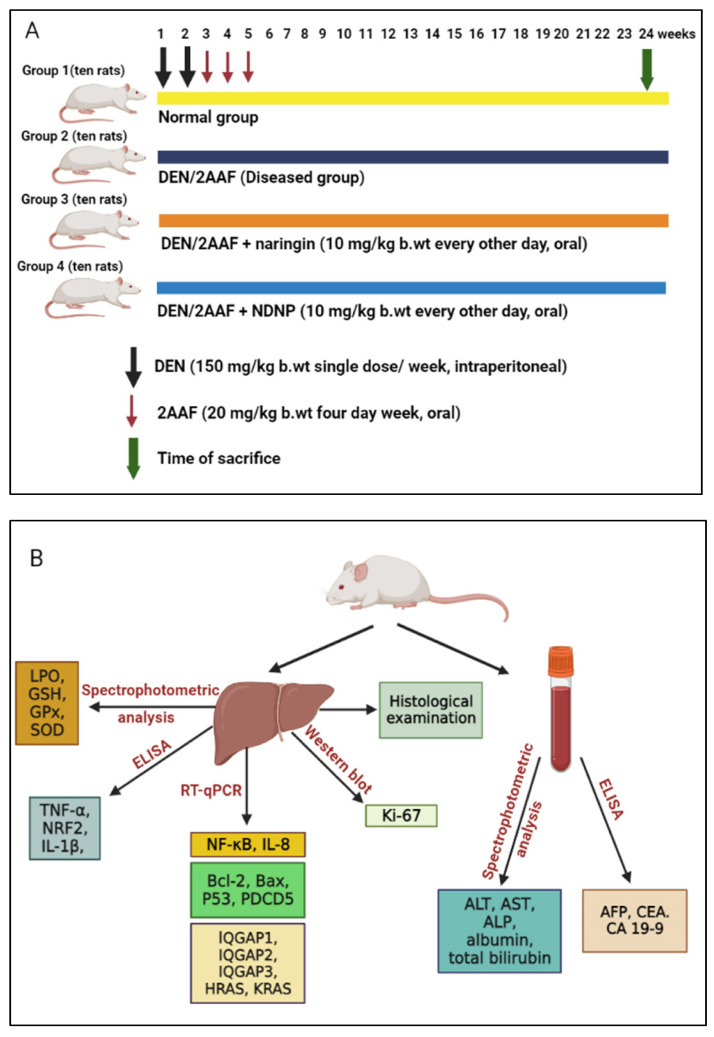
Schematics of the experimental design and animal grouping: Wistar adult male rats were divided into four groups and the number of animals in each group was ten (**A**); blood, tissue sampling, and techniques (**B**).

Rats were divided into four groups of ten (Figure 8A): an untreated normal group (group I) and three groups administered 150 mg/kg body weight (b.w.) DEN twice weekly for two weeks, followed by oral gavage administration of 20 mg/kg b.w. 2AAF four times/week for three weeks [29]. Group II served as a positive control group and received DEN/2AAF and a vehicle for 24 weeks, while groups III and IV received 10 mg/kg b.w. free naringin or NDNP per day by oral gavage in a 5 mL 1% carboxymethyl cellulose vehicle for 24 weeks [21]. Groups I and II received equal volumes of the vehicle during the free naringin or NDNP treatment period.

### 3.5. Blood and Liver Tissue Sampling

After 24 weeks, animals given a light anesthetic with diethyl ether were sacrificed. Blood samples were taken and, after clotting, centrifuged at 3000 r.p.m. for 15 min. Sera were collected in sterile tubes and kept at −20 °C to measure liver function and tumor marker parameters. After decapitation by head dislocation, the liver was excised from each animal, perfused in sterile saline (0.9% NaCl), and blotted on filter paper to remove the saline. Four pieces of each liver were taken. One liver sample was washed with ice-cooled sterile saline solution (0.9% NaCl) and homogenized in 10 mL 0.9% NaCl to yield 1% homogenate (*w*/*v*). Liver homogenates were centrifuged at 3000 rpm for 15 min. The supernatants were stored at −30 °C until they were used in the investigation of antioxidant activities, oxidative stress, and the levels of inflammatory biomarkers; i.e., TNF-α, IL-1β, NRF2. The second liver portion was kept at −70 °C in a sterilized tube and used for RNA isolation and real-time (RT) polymerase chain reaction (PCR) assays. A third liver tissue sample was also kept at −70 °C and used for Western blot analysis of Ki-67. The fourth liver portion was processed for sectioning and staining with haematoxylin and eosin (H&E) for histopathological examination after incubation for one day in 10% neutral buffered formalin for fixation (Figure 8B).

### 3.6. Detection of Serum Liver Function Parameters

ALT and AST activities were determined according to the method described by Gella et al. [30]. Activity of ALP was determined according to the methods described by Schumann et al. [31]. Levels of total bilirubin and albumin were estimated according to Jendrassik [32] and Doumas et al. [33], respectively. Each of the reagent kits used was purchased from HUMAN Gesellschaft fur Biochemica und Diagnostica mbH, Max-Planck-Ring 21, 65205 Wiesbaden, Germany.

### 3.7. Detection of Tumor Markers Levels in Serum

The serum levels AFP, CEA, and CA19.9 were measured using ELISA kits from R&D Systems (Minneapolis, MN, USA) in accordance with the manufacturer’s instructions.

### 3.8. Detection of Hepatic Oxidative Stress and Antioxidant Status Estimation

Liver LPO, GSH content, and GPx, and SOD activities were analyzed using kits from Bio-diagnostic (Dokki, Giza, Egypt).

### 3.9. Detection of Hepatic TNF-α, IL1β, and NRF2

Liver TNF-α, IL-1β, and NRF2 were assayed according to the manufacturer’s instructions using ELISA kits purchased from R&D Systems (Minneapolis, MN, USA).

### 3.10. Isolation of Total RNA and Reverse Transcription Quantitative PCR (RT-qPCR)

Total RNA from the liver was isolated using Trizol (Invitrogen, 1600 Faraday Ave Carlsbad, CA, 92008-7313, USA) according to the method described by Sthoeger et al. [34]. Following the completion of the isolation procedure in accordance with the manufacturer’s instructions, the 260:280 ratios were measured to assess the quality of the RNA. The production of complementary DNA (cDNA) was carried out using a high-capacity cDNA reverse transcription kit (Applied Biosystems, Foster City, CA 94404, USA). The primers employed in these tests are displayed in Table 4. The RT-qPCR data were analyzed utilizing the relative gene expression approach (i.e., ΔΔCT), as detailed in the Applied Biosystems User Bulletin No. 2. The β-actin gene was used to normalize each sample and gene.

### 3.11. Western Blot Analysis

A clear supernatant was obtained after centrifuging liver samples that had been homogenized in radioimmuno precipitation assay (RIPA) buffer. The total protein content was measured using a Bradford assay kit (SK3041; Bio Basic Inc., Markham, ON L3R 8T4 Canada) following He [35]. Then, 30 µg of proteins was transferred to nitrocellulose membranes after being separated using sodium dodecyl sulfate–polyacrylamide gel electrophoresis (SDS-PAGE) (Bio-Rad Laboratories Inc, 1000 Alfred Nobel Drive Hercules, CA, USA). The SDS-PAGE TGX Stain-Free FastCast was prepared according to the manufacturer’s instructions. The gel was assembled in a transfer sandwich from below to above (filter paper, PVDF membrane, gel, and filter paper). The sandwich was placed in the transfer tank with 1x transfer buffer, which was composed of 25 mM Tris, 190 mM glycine, and 20% methanol. Then, the blot was run for 7 min at 25 V to allow protein bands to be transferred from gel to membrane using BioRad Trans-Blot Turbo. The membranes were incubated overnight at 4 °C with the corresponding primary antibodies against Ki-67 and β-actin primary antibodies (1:1000 dilution) in 5% nonfat milk in Tris-buffered saline with Tween 20 (TBST) [36,37]. The membranes were then developed using the chemiluminescent substrate (Clarity™ Western ECL substrate Bio-Rad) and probed with the corresponding horseradish peroxidase (HRP)-conjugated secondary antibodies (goat anti-rabbit IgG HRP, 1 mg goat monoclonal antibody, Novus Biologicals, 10730 E Briarwood Ave Ste 100, Englewood, CO, 80112, USA). Finally, image analysis software was used to compare the band intensities of the target proteins normalized to that of the housekeeping protein β-actin with the intensity ratio in the control sample using the ChemiDoc MP imager.

### 3.12. Histopathological Investigation

Pieces of the liver were fixed in neutral buffered formalin 10% for 24 h following treatment with the paraffin-embedding method, dehydration with increasing concentrations of ethyl alcohol, xylene clearing, paraffin submersion, and coating with paraffin wax at 60 °C. Sections were cut to 4 microns thickness using a slide microtome and stained with haematoxylin and eosin (H&E) according to the method described in [38]. The examination was carried out using an electric light microscope.

### 3.13. Statistical Analysis

Results are shown as means ± standard error (SE). One-way analysis of variance (ANOVA) was used to compare the statistical differences between the groups. Duncan’s method for post hoc analysis (SPSS version 20 software) was used to compare different groups, with significance set at *p* < 0.05.

## 4. Discussion

HCC is the third-leading cause of cancer-related death worldwide [39]. Naringin is a natural flavonoid found in grapefruit that is included in many Chinese herbal medicines due to its wide range of potentially beneficial bioactivities [40]. However, these raw compounds may have therapeutic limitations similar to synthetic compounds; in particular, poor tissue targeting and concomitant side effects. Therefore, much research is currently devoted to developing nanoformulations based on natural products to treat various human diseases, including cancer [24], with superior bioavailability and limited effects on nontarget tissue. Here, we investigated whether the nanoformulation of naringin has more potent effects than free naringin against DEN-induced HCC via an assessment of the anticarcinogenic, antioxidant, antiinflammatory, and antiproliferative efficacies.

Administration of DEN/2AAF induced substantial hepatocellular damage, as evidenced by increased serum ALT AST, ALP, and total bilirubin, as well as reduced serum albumin, consistent with previous research [29,41]. In addition to acute impairment of hepatic functions, this liver injury can activate the neoplastic process [42]. Elevations in serum ALT and AST are due to leakage from damaged hepatocytes [36]. Similarly, total bilirubin is released due to nonspecific changes in hepatocyte plasma membrane integrity and/or permeability, while ALP elevation results from pathological alterations in biliary flow [42]. All of these increases were reversed by naringin and NDNP, suggesting that these compounds are able to prevent loss of membrane integrity in addition to their effects on the preservation of hepatic functions, such as albumin synthesis [43]. Notably, the nanoformula was more potent in improving liver function and structural integrity than free naringin, likely due to improved bioavailability in liver. In support of this attribution, it was stated by Ratheesh et al. that nanodrug delivery helps to increase the bioavailability of drugs and, thereby, can be used to specifically target cells and tissues [44]. In addition, in our previous work [27], it was found that the cumulative release of naringin–dextrin nanoformula demonstrated higher release and a more sustained release pattern compared to free naringin.

The gold standard diagnostic marker for HCC is serum AFP [45], while the combination of AFP, CA19-9, and CEA may help with the diagnosis of primary hepatic cancer [46]. In the current investigation, serum AFP, CEA, and CA19-9 were markedly elevated by DEN/2AAF administration compared to the normal control group, in accordance with Ahmad et al. [29] and Mansour et al. [47]. Both AFP and CEA are released by tumors, and increased serum CEA is associated with larger tumors and metastasis [48]. Additionally, serum CA19-9 is elevated in a small proportion of patients with HCC [49]. At 24 weeks after the DEN/2AAF treatment period, liver cancers were present in all animals in the DEN control group as revealed by histological examination. Histopathological analysis of these HCC model animals also showed disruption of the normal lobular structure and tumor cells organized in glandular and lamellar forms, with clear deeply eosinophilic foci. Both naringin and NDNP reversed the serum elevations in AFP, CEA, and CA19-9, but NDNP was more potent than free naringin, suggesting greater antitumor efficacy, a notion confirmed by histopathological analysis of liver tissue sections.

DEN is a strong environmental carcinogen that causes cellular transformation by producing ROS that damage proteins, lipids, and DNA [50,51]. Numerous studies have shown that DNA damage and mutations caused by excessive ROS production and ensuing oxidative stress is the key event in hepatocarcinogenesis [52,53]. Rats treated with DEN demonstrated an obvious redox imbalance in liver tissue, with significant decreases in GPx, SOD activity, and GSH content and a significant increase in hepatic LPO, consistent with previous studies by Ahmed et al. [29] and Mo’men et al. [54]. Further, liver LPO production in rats is believed to reflect the degree of cell damage [53].

**Figure 9:**
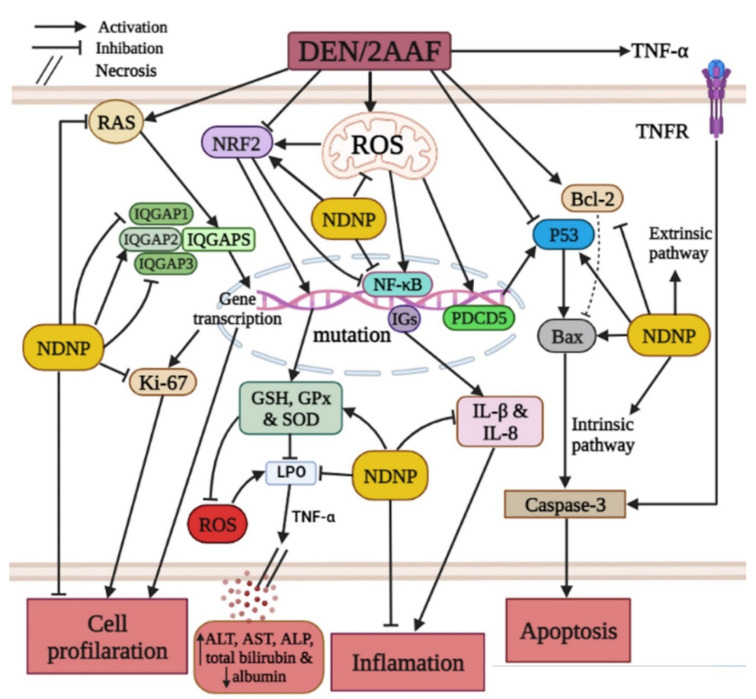
Schematic diagram showing the hepatoprotective mechanisms of naringin–dextrin nanoparticles against DEN/2AAF: suppression of oxidative stress, inflammation, and proliferation, as well as induction of transformed cell apoptosis. DEN: diethylnitrosamine; 2AAF: 2-acetylaminofluorene; ROS: reactive oxygen species; IGs: inflammatory genes; IQGAP1: isoleucine–glutamine motif-containing GTPase-activating protein 1; IQGAP2: isoleucine–glutamine motif-containing GTPase-activating protein 2; IQGAP3: Isoleucine–glutamine motif-containing GTPase-activating protein 3; NF-κB: nuclear factor-kappa B; IL-8: interleukin-8; IL-1β: interleukin-1β; Bcl-2: B-cell lymphoma-2; Bax: Bcl-2-associated X protein; p53: tumor suppressor protein 53; PDCD5: programmed cell death 5. RAS: rat sarcoma viral oncogene homolog; ALT: alanine transaminase; AST: aspartate transaminase; ALP: alkaline phosphatase; NDNP: naringin-dextrin nanoparticle; TNF-α: tumor necrosis factor alpha; TNFR: tumor necrosis factor alpha receptor; NRF2: nuclear factor E2-related factor 2; LPO: lipid peroxidation; GSH: Glutathione; SOD: Superoxide dismutase; GPx: glutathione peroxidase.

Again, both naringin and NDNP reversed these effects. Naringin has well-documented efficacy against LPO [55]. Acharya et al. [43] also reported that naringin dose-dependently reduced oxidative damage markers, including the LPO marker MDA, consistent with free radical scavenging activity. This suppression of ROS accumulation and enhanced antioxidant capacity also prevents carcinogens from being metabolically activated [56] and decreases ROS-induced mtDNA damage (Figure 9) [57,58]. Administration of NDNP enhanced antioxidant defenses in rat liver more effectively than free naringin at the same dose, again suggesting that this nanoformulation enhances liver bioavailability and antioxidant efficacy.

Treatment with DEN/2AAF also elevated total levels of TNF-α, IL-1β, NF-κB, and IL-8, while reducing NRF2 levels, which was in accordance with published studies [36,59,60] and suggests that chronic inflammation may contribute to DEN-induced hepatic oxidative stress and tissue damage (Figure 9). The molecular and cellular processes that start with liver injury and end with fibrosis and HCC are triggered by the release of TNF-α, IL-1β, and IL-8, among other proinflammatory factors [61]. The levels of TNF-α and IL-1β are controlled by NF-κB and, subsequently, enhance vascular permeability, cell proliferation, and inflammation [62]. According to Ahmed et al. [63], excessive TNF-α production contributes to the pathogenesis of several inflammatory diseases by causing and maintaining inflammation. NRF2 deficiency increases inflammatory responses by activating NF-κB (Figure 9) and ensuing proinflammatory process [64]. NF-κB also regulates the transcription of more than 150 genes involved in cell proliferation, migration, invasion, survival, and apoptosis escape [65]; thus, overexpression is strongly linked to cancer development. Further, NF-κB expression is activated in response to oxidative stress [66] and is strongly associated with more aggressive cancer [67].

Thus, naringin and NDNP likely reduced the inflammatory response by suppressing oxidative stress-induced NF-κB expression (Figure 9). Consistent with these findings, Shirani et al. [68] and Lv et al. [69] reported that naringin treatment dramatically reduced the expression levels of inflammatory mediators, such as TNF-α, IL-8, NF-κB, and IL-1β, and elevated NRF2. Naringin prevented inflammation, apoptosis, autophagy, and oxidative DNA damage by reducing TNF-α and IL-1β levels and NF-κB expression [64]. A recent study also found that naringin alleviated liver injury by activating NRF2 and decreasing TNF-α and NF-κB expression [69]. Manna et al. [70] and Dong et al. [71] similarly reported that naringin diminished liver injury by reducing the generation of proinflammatory mediators and by increasing NRF2 levels, thereby reducing NF-κB expression and the production of downstream inflammatory cytokines [69]. The naringin-derived NPNP was even more effective, in accordance with several previous reports that natural product-derived nanomedicines can be effective treatments for cancer and inflammatory disorders [72,73].

Apoptosis is regulated primarily by two pathways [74]: the death receptor-dependent extrinsic pathway and the mitochondria-dependent intrinsic pathway (Figure 9). Rats treated with DEN/2AAF showed increase expression of antiapoptotic Bcl-2 and reduced expression of proapoptotic Bax compared to controls, in agreement with previous reports [29,75,76]. The antiapoptotic Bcl-2 regulates apoptosis by antagonizing the function of proapoptotic Bax (Figure 9) [77]. According to Rückert et al. [78] tumors generally show elevated Bcl-2 expression, so hepatic Bcl-2 upregulation in the DEN/2AAF group suggests that malignant cells were resistant to apoptosis, possibly due to increased gene copy numbers, transcription, and (or) translation of Bcl-2, as observed for other cancer-related genes [79,80]. DEN also decreased the expression of Bax in the liver of cancerous rats [76,81]. Additionally, rats administered DEN exhibited reduced hepatic expressions of p53 and PDCD5 compared to controls, which may be necessary for resistance to apoptosis and DEN-induced carcinogenesis. XinYou et al. [74] and Zoheir et al. [60] concluded that diminished expression of p53 in HCC resulted from downregulation of PDCD5. Further, p53 downregulation is among the most prevalent genetic changes in HCC [29]. Recently, it was discovered that PDCD5 translocates to the nucleus in response to DNA damage and then interacts with p53 to encourage apoptosis [82]. DEN treatment may enhance resistance to apoptosis by inducing oxidative stress and disrupting signaling pathways that control the transcription of apoptotic genes [83].

Alternatively, naringin and NDNP treatment may have enhanced sensitivity to apoptosis by lowering Bcl-2 expression and increasing Bax, p53, and PDCD5 expressions. Consistent with this notion, Xie et al. [84] reported that naringin downregulated Bcl-2 expression and upregulated Bax expression to trigger apoptosis, as p53 is a proapoptotic mediator and a regulator of the cell cycle and apoptosis. While PDCD5 can accelerate apoptosis in various cell types [85,86], elevated Bax, p53, and PDCD5 expression and reduced Bcl-2 expression may mediate the antiproliferative and proapoptotic actions of naringin and NDNP, thereby diminishing the survival of HCC cells (Figure 9). Indeed, naringin was shown to enhance apoptosis and reduce cell proliferation in the Wistar rat DEN-induced model of liver carcinogenesis [87]. Most clinical chemotherapy drugs suppress tumor growth by inducing cancer cell apoptosis, and HCC may be vulnerable to these effects [88]. Mohamed et al. [27] further reported that naringin and NDNP induced apoptosis of HCC cells and confirmed the stronger effect of NDNP.

The IQGAPs are a family of highly conserved multidomain proteins that regulate cell migration and adhesion, and various cytokine signaling pathways, among other functions [89]. Wistar rats that received DEN treatment showed increased expression of IQGAP1 and IQGAP3 but reduced expression of IQGAP2 compared to normal rats. Similarly, Zoheir et al. [90] reported that IQGAP1 was upregulated while IQGAP2 was downregulated by DEN. Cancer development may be influenced by IQGAP1, as this factor controls numerous cellular processes relevant to cancer development. Further, although there are no mutations, several cancer types exhibit an unusually high expression of IQGAP1 [91]. Both IQGAP1 and IQGAP3 are considered oncogenes in HCC and, thus, can serve as highly sensitive and specific biomarkers for this type of tumor, while IQGAP2 may act as a tumor suppressor [92,93]. Several studies have reported higher expression of IQGAP3 in patients with HCC [94,95,96], and Shi et al. [97] concluded that IQGAP3 promotes tumor metastasis and the epithelial–mesenchymal transition in patients with HCC. Wu et al. [98] found that IQGAP3 promotes the malignant progression of tumors by increasing the ability of HCC cells to invade and spread. Thus, IQGAP3 is considered a potential therapeutic target to combat metastasis and tumor progression [97]. DEN acts as a carcinogen by inducing uncontrolled cell proliferation and hepatocellular necrosis [99], as well as ROS production and ensuing DNA damage [100]. Naringin and NDNP reduced hepatic IQGAP1 and IQGAP3 expression and elevated IQGAP2 expressions compared to rats receiving DEN/2AAF alone. These findings are consistent with Mohamed et al. [27], who reported that naringin and NDNP have anticancer and antiproliferation effects in human liver cancer cells and that the nanostructure was more effective than free naringin.

RAS proteins serve as molecular switches that participate in a number of intracellular signal transduction pathways, such as the MAPK and PI3K signaling pathways, which are essential for cell growth, differentiation, apoptosis, cellular adhesion, cell migration, and microtubule integrity [101,102]. The expressions of HRAS and KRAS were higher in DEN/2AAF-treated rats compared to normal rats, in accordance with Zoheir et al. [103], who reported significantly upregulated HRAS and KRAS mRNA expressions in an animal model of DEN-induced hepatic cancer. Both HRAS and KRAS are frequently overexpressed in other cancer types as well [102]. Treatment with naringin or NDNP reversed these increases, consistent with clinical findings by Mohamed et al. [27], and again, NDNP was more effective than free naringin.

Excessive cell proliferation is the core feature of cancer and proliferation rate is a major predictor of prognosis and treatment efficacy [104]. Administration of DEN dramatically increased the expression of the proliferation marker Ki-67, consistent with previous studies [29,53], which found elevated Ki-67 expression in HCC (Figure 9). Naringin and NDNP treatment have been reported to reduce Ki-67 expression [105,106]. In addition, naringin increased apoptosis and decreased proliferation in rat liver cancer cells [107]. We conclude that NDNP enhanced antioxidant defenses, thereby preventing oxidative stress, and suppressed inflammation, apoptosis resistance, and hyperproliferation of liver cells in response to DEN/2AAF treatment more effectively than naringin in a rat model of HCC. Thus, NDNP may provide a more efficacious treatment for HCC. However, the current research has several limitations, including the absence of techniques such as Western blot and direct immunohistochemical analysis of apoptosis, cell signaling, and proliferation biomarkers. In addition, it is essential to compare the efficacy of NDNP to current drugs used against HCC.

## 5. Conclusions

Dextrin possesses many functional attributes suitable for producing nanocarriers. In an animal model of hepatocellular carcinoma, naringin–dextrin nanoparticles suppressed DEN/2AAF-induced HCC with greater efficacy than free naringin by reducing oxidative stress, inflammation, and tumor cell proliferation while enhancing apoptosis. We suggest that NDNP could be a prototype for a new family of targeted drugs with greater clinical efficacy than the free parent compound. Further, the use of natural compounds in these nanoformulae may enhance pharmacological responses, resulting in better patient outcomes. However, further studies are required to assess and compare the effects of naringin and NDNP on human HCC xenografts and clinical studies are also required to assess the safety and efficacy of these agents in human beings.

## Figures and Tables

**Table 1:** Effect of naringin and NDNP on serum parameters related to liver function.

Groups	ALT	AST	ALP	Total Bilirubin	Albumin
	(U/L)	(U/L)	(U/L)	(mg/dL)	(g/dL)
Normal group	40.40 ± 3.05	97.00 ± 1.52	206.08 ± 6.45	0.27 ± 0.02	3.75 ± 0.11
DEN/2AAF-CG	70.18 ± 2.68 ^+^	161.66 ± 2.96 ^+^	506.28 ± 11.46 ^+^	0.90 ± 0.07 ^+^	2.88 ± 0.05 ^+^
DEN/2AAF + naringin	50.31 ± 1.31 ^+^*	111.16 ± 4.24 ^+^*	398.66 ± 10.59 ^+^*	0.48 ± 0.03 ^+^*	3.32 ± 0.07 ^+^*
DEN/2AAF + NDNP	41.36 ± 1.73 *^#^	100.50 ± 2.51 *^#^	338.6 ± 10.69 ^+^*^#^	0.40 ± 0.04 *	3.55 ± 0.04 *^#^

Data are expressed as means ± standard error. Number of detected samples in each group was six. ^+^ Significant compared to normal group; * significant compared to DEN/2AAF-CG; and ^#^ significant compared to DEN/2AAF + naringin group at *p* < 0.05. CG: control group; NDNP: naringin-dextrin nanoparticle; ALT: alanine transaminase; AST: aspartate transaminase; ALP: alkaline phosphatase.

**Table 2:** Effect of naringin and NDNP on liver TNF-α, IL-1β, and NRF2 levels in DEN/2AAF-administered rats.

Groups	TNF-α	IL-1β	NRF2
	(Pg/mg Tissue)	(Pg/mg Tissue)	(Pg/mg Tissue)
Normal group	22.53 ± 1.04	35.64 ± 1.07	259.41 ± 2.81
DEN/2AAF- CG	126.66 ± 0.93 ^+^	165.78 ± 0.80 ^+^	93.80 ± 0.77 ^+^
DEN/2AAF + naringin	55.23 ± 0.78 ^+^*	74.13 ± 0.74 ^+^*	219.41 ± 1.2 ^+^*
DEN/2AAF + NDNP	34.00 ± 0.77 ^+^*^#^	55.41 ± 0.59 ^+^*^#^	236.51 ± 3.12 ^+^*^#^

Data are expressed as means ± standard error. Number of detected samples in each group was six. ^+^ Significant compared to normal group; * significant compared to DEN/2AAF-CG; and ^#^ significant compared to DEN/2AAF + naringin group at *p* < 0.05. CG: control group. NDNP: naringin-dextrin nanoparticle; TNF-α: tumor necrosis factor alpha; IL-1β: interleukin 1 beta: NRF2: nuclear factor E2-related factor 2.

**Table 3:** Histopathological scores of liver lesions in normal control group, DEN/2AAF-administered group, and DEN/2AAF-administered groups supplemented with naringin and NDNP.

Histopathological Changes	Score	Normal	DEN/2AAF	DEN/2AAF+ Naringin	DEN/2AAF+ NDNP
Karyomegalic hepatocytic nuclei with more than one prominent nucleoli	0	6(100%)	-	5(83.3%)	6(100%)
	I	-	-	1(16.7%)	-
	II	-	1(16.7%)	-	-
	III	-	5(83.3%)	-	-
Steatosis and cytoplasmic vacuolization of hepatocyte	0	6(100%)	-	2(33.3%)	4(66.6%)
	I	-	-	3 (50%)	1(16.7%)
	II	-	2(33.3%)	1(16.7%)	1(16.7%)
	III	-	4 (66.6%)	-	-
Dysplastic hepatocytes with mitotic figures	0	6(100%)	-	4(66.6%)	6(100%)
	I	-	-	2(33.3%)	-
	II	-	3(50%)	-	-
	III	-	3(50%)	-	-
Clear hepatocellular focus	0	6(100%)	-	1(16.7%)	6(100%)
	I	-	-	4(66.6%)	-
	II	-	-	1(16.7%)	-
	III	-	6 (100%)	-	-
Deeply eosinophilic foci of hepatocytes	0	6(100%)	-	2(100%)	5(83.8%)
	I	-	1(16.7%)	3(50%)	1(16.6%)
	II	-	2(33.3%)	1(16.7)	-
	III	-	3(50%)	-	-
Inflammatory cell infiltration	0	6(100%)	-	3(50%)	4(66.6%)
	I	-	-	3(50%)	2(33.3%)
	II	-	1(16.7%)	-	-
	III	-	5(83.3%)	-	-

0: Null; I: mild; II: moderate; III: severe lesions. The number of animals in each group was 6. The percentages in parentheses are the percentages of animals for each grade of lesion.

**Table 4:** Primer sequences for rats.

Genes	GenBank Accession Number	Sequence (5′–3′)
IQGAP1	NM_001108489.1	F: GCGGCTTCCAACAAGATGTTT R: CAGCAGTTCATGGATGGGGT
IQGAP2	XM_039103456.1	F: GGAATCTACGGACGCTGGAG R: CACAGTACTGGGTGTGTCCC
IQGAP3	NM_001191709.1	F: AGCCTATGATCGTCTCACAGC R: CACAGGTACTGGTAGGCGAC
NF-κB	NM_001276711.1	F: TTCAACATGGCAGACGACGA R: TGCTCTAGTATTTGAAGGTATGGG
IL-8	X77797.1	F: CAGAGACTTGGGAGCCACTC R: CAGAGTAAAGGGCGGGTCAG
Bcl-2	NM_016993.1	F: TAAGCTGTCACAGAGGGGCT R: TGAAGAGTTCCTCCACCACC
Bax	NM_007527.3	F: CTGGATCCAAGACCAGGGTG R: CCTTTCCCCTTCCCCCATTC
P53	NM_030989.3	F: GTTTTTGTTCCTGAGCCCCG R: GAGCAAGGGGTGACTTTGGG
PDCD5	NM_001106247.1	F: TGAAGCGATTCCAACCGAGT R: GCTCCGTGGGTCTGTCTAAG
HRAS	NM_ 001130441.1	F: CTGTCCTGACACCAGGCTC R: ATGGACCCTCCTGTAGCCAT
KRAS	NM_ 031515.3	F: AGACACGAAACAGGCTCAGG R: GCATCGTCAACACCCTGTCT
β-actin	NM_031144.3	F: TCACTATCGGCAATGTGCGG R: GCTCAGGAGGAGCAATGATG

IQGAP1: isoleucine–glutamine motif-containing GTPase-activating protein 1; IQGAP2: isoleucine–glutamine motif-containing GTPase-activating protein 2; IQGAP3: isoleucine–glutamine motif-containing GTPase-activating protein 3; NF-κB: nuclear factor-kappa B; IL-8: interleukin-8. Bcl-2: B-cell lymphoma-2; Bax: Bcl-2-associated X protein; p53: tumor suppressor protein 53; PDCD5: programmed cell death 5. HRAS: Harvey rat sarcoma viral oncogene homolog; KRAS: Kirsten rat sarcoma viral oncogene homolog.

## Data Availability

Data is contained within the article.

## Supplementary Materials

The following supporting information can be downloaded at: https://www.mdpi.com/article/10.3390/ph15121558/s1.

## References

[1] Saber S., Mahmoud A., Goda R., Helal N.S., El-Ahwany E., Abdelghany R.H. (2018). Perindopril, fosinopril and losartan inhibited the progression of diethylnitrosamine-induced hepatocellular carcinoma in mice via the inactivation of nuclear transcription factor kappa-B. Toxicol. Lett..

[2] Saber S., Khodir A.E., Soliman W.E., Salama M.M., Abdo W.S., Elsaeed B., Nader K., Abdelnasser A., Megahed N., Basuony M. (2019). Telmisartan attenuates N-nitrosodiethylamine-induced hepatocellular carcinoma in mice by modulating the NF-κB-TAK1-ERK1/2 axis in the context of PPARγ agonistic activity. Naunyn-Schmiedeberg’s Arch. Pharmacol..

[3] Younis N.S., Ghanim A.M., Saber S. (2019). Mebendazole augments sensitivity to sorafenib by targeting MAPK and BCL-2 signalling in n-nitrosodiethylamine-induced murine hepatocellular carcinoma. Sci. Rep..

[4] Seydi E., Motallebi A., Dastbaz M., Dehghan S.G., Salimi A., Nazemi M., Pourahmad J. (2015). Selective toxicity of Persian Gulf sea cucumber (*Holothuria parva*) and sponge (*Haliclona oculata*) methanolic extracts on liver mitochondria isolated from an animal model of hepatocellular carcinoma. Hepat Mon..

[5] Asafo-Agyei K.O., Samant H. (2021). Hepatocellular Carcinoma.

[6] Sivalingam K., Amirthalingam V., Ganasan K., Huang C.Y., Viswanadha V.P. (2019). Neferine suppresses diethylnitrosamine-induced lung carcinogenesis in Wistar rats. Food Chem. Toxicol..

[7] Hidajat M., McElvenny D.M., Ritchie P., Darnton A., Mueller W., Agius R.M., Cherrie J.W., De Vocht F. (2020). Lifetime cumulative exposure to rubber dust, fumes and N-nitrosamines and non-cancer mortality: A 49-year follow-up of UK rubber factory workers. Occup. Environ. Med..

[8] Arboatti A.S., Lambertucci F., Sedlmeier M.G., Pisani G., Monti J., Álvarez M.D., Frances D.E., Ronco M.T., Carnovale C.E. (2018). Diethylnitrosamine increases proliferation in early stages of hepatic carcinogenesis in insulin-treated type 1 diabetic mice. BioMed Res. Inter..

[9] Alsahli M.A., Almatroodi S.A., Almatroudi A., Khan A.A., Anwar S., Almutary A.G., Alrumaihi F., Rahmani A.H. (2021). 6-Gingerol, a major ingredient of ginger attenuates diethylnitrosamine-induced liver injury in rats through the modulation of oxidative stress and anti-inflammatory activity. Mediat. Inflame.

[10] Senthil K., Aranganathan S., Nalini N. (2004). Evidence of oxidative stress in the circulation of ovarian cancer patients. Clin. Chim. Acta.

[11] Panieri E., Santoro M.M. (2016). ROS homeostasis and metabolism: A dangerous liaison in cancer cells. Cell Death Dis..

[12] Schumacker P.T. (2006). Reactive oxygen species in cancer cells: Live by the sword, die by the sword. Cancer Cell.

[13] Tornesello M.L., Buonaguro L., Izzo F., Buonaguro F.M. (2016). Molecular alterations in hepatocellular carcinoma associated with hepatitis B and hepatitis C infections. Oncotarget.

[14] Okada M., Shibuya K., Sato A., Seino S., Watanabe E., Suzuki S., Kitanaka C. (2013). Specific role of JNK in the maintenance of the tumor-initiating capacity of A549 human non-small cell lung cancer cells. Oncol. Rep..

[15] Zhao H., Wu L., Yan G., Chen Y., Zhou M., Wu Y., Li Y. (2021). Inflammation and tumor progression: Signaling pathways and targeted intervention. Signal Transduct. Target Ther..

[16] Carneiro B.A., El-Deiry W.S. (2020). Targeting apoptosis in cancer therapy. Nat. Rev. Clin. Oncol..

[17] Ryan M.B., Corcoran R.B. (2018). Therapeutic strategies to target RAS-mutant cancers. Nat. Rev. Clin. Oncol..

[18] Diepstraten S.T., Anderson M.A., Czabotar P.E., Lessene G., Strasser A., Kelly G.L. (2022). The manipulation of apoptosis for cancer therapy using BH3-mimetic drugs. Nat. Rev. Cancer.

[19] Adiban H., Shirazi F.H., Gholami S., Kamalinejad M., Hosseini S.H., Noubarani M., Eskandari M.R. (2019). Chemopreventive effect of quince (*Cydonia oblonga* Mill.) fruit extract on hepatocellular carcinoma induced by diethylnitrosamine in rats. Inter. Pharm. Acta..

[20] Shirazi F.H., Piri M., Keshavarz S., Gholami S., Hosseini S.H., Noubarani M., Andalib S., Kamalinejad M., Adiban H., Eskandari M.R. (2018). Olive fruit (*Olea europaea L.):* Chemopreventive effect in the rat model of hepatocellular carcinoma. Pharma. Nut..

[21] Camargo C.A., Gomes-Marcondes M.C., Wutzki N.C., Aoyama H. (2012). Naringin inhibits tumor growth and reduces interleukin-6 and tumor necrosis factor α levels in rats with Walker 256 carcinosarcoma. Anticancer Res..

[22] Sun J., Kormakov S., Liu Y., Huang Y., Wu D., Yang Z. (2018). Recent progress in metal-based nanoparticles mediated photodynamic therapy. Molecules.

[23] Wu C., Chen Z., Hu Y., Rao Z., Wu W., Yang Z. (2018). Nanocrystals: The preparation, precise control and application toward the pharmaceutics and food industry. Curr. Pharm. Des..

[24] Kashyap D., Tuli H.S., Yerer M.B., Sharma A., Sak K., Srivastava S., Pandey A., Garg V.K., Sethi G., Bishayee A. (2021). Natural product-based nanoformulations for cancer therapy: Opportunities and challenges. Semin. Cancer Biol..

[25] Zhang N.N., Yu R.S., Xu M., Cheng X.Y., Chen C.M., Xu X.L., Lu C.Y., Lu K.J., Chen M.J., Zhu M.L. (2018). Visual targeted therapy of hepatic cancer using homing peptide modified calcium phosphate nanoparticles loading doxorubicin guided by T1 weighted MRI. Nanomed. Nanotech. Biol. Med..

[26] Shamay Y., Shah J., Işık M., Mizrachi A., Leibold J., Tschaharganeh D.F., Roxbury D., Budhathoki-Uprety J., Nawaly K., Sugarman J.L. (2018). Quantitative self-assembly prediction yields targeted nanomedicines. Nat. Mater..

[27] Mohamed E.E., Abdel-Moneim A., Ahmed O.M., Zoheir K.M., Eldin Z.E., El-Shahawy A.A. (2022). Anticancer activity of a novel naringin–dextrin nanoformula: Preparation, characterization, and in vitro induction of apoptosis in human hepatocellular carcinoma cells by inducing ROS generation, DNA fragmentation, and cell cycle arrest. J. Drug Deliv. Sci. Tech..

[28] Manchun S., Dass C.R., Sriamornsak P. (2014). Designing nanoemulsion templates for fabrication of dextrin nanoparticles via emulsion cross-linking technique. Carbohy. Polym..

[29] Ahmed O.M., Fahim H.I., Mohamed E.E., Abdel-Moneim A. (2022). Protective effects of *Persea americana* fruit and seed extracts against chemically induced liver cancer in rats by enhancing their antioxidant, anti-inflammatory, and apoptotic activities. Environ. Sci. Pollut. Res..

[30] Gella F.J., Olivella T., Cruz P.M., Arenas J., Moreno R., Durban R., Gomez J.A. (1985). A simple procedure for routine determination of aspartate aminotransferase and alanine aminotransferase with pyridoxal phosphate. Clin. Chim. Acta.

[31] Schumann G., Bonora R., Ceriotti F., Férard G., Ferrero C., Franck P.F., Gella F.J., Hoelzel W., Jørgensen P.J., Kanno T. (2002). IFCC primary reference procedures for the measurement of catalytic activity concentrations of enzymes at 37 °C. Part 6: Reference procedure for the measurement of catalytic concentration of γ-Glutamyltransferase. Clin. Chem. Lab. Med..

[32] Jendrassik L. (1938). Colorimetric determination of bilirubin. Biochemistry.

[33] Doumas B.T., Watson W.A., Biggs H.G. (1971). Albumin standards and the measurement of serum albumin with bromcresol green. Clin. Chim. Acta.

[34] Sthoeger Z., Zinger H., Sharabi A., Asher I., Mozes E. (2013). The tolerogenic peptide, hCDR1, down-regulates the expression of interferon-α in murine and human systemic lupus erythematosus. PLoS ONE.

[35] He F. (2011). Bradford Protein Assay. Bio-Protocol.

[36] Tawfik N.G., Mohamed W.R., Mahmoud H.S., Alqarni M.A., Naguib I.A., Fahmy A.M., Ahmed O.M. (2022). Isatin Counteracts Diethylnitrosamine/2-Acetylaminofluorene-Induced Hepatocarcinogenesis in Male Wistar Rats by Upregulating Anti-Inflammatory, Antioxidant, and Detoxification Pathways. Antioxidant.

[37] Yassin N., AbouZid S.F., El-Kalaawy A.M., Ali T.M., Elesawy B.H., Ahmed O.M. (2021). Tackling of renal carcinogenesis in Wistar rats by Silybum marianum total extract, silymarin, and silibinin via modulation of oxidative stress, apoptosis, Nrf2, PPAR, NF-κB, and PI3K/Akt signaling pathways. Oxidative Med. Cell. Longev..

[38] Bancroft J.D., Gamble M. (2008). Theory and practice of histological techniques. Elsevier Health Sciences.

[39] Sperandio R.C., Pestana R.C., Miyamura B.V., Kaseb A.O. (2022). Hepatocellular carcinoma immunotherapy. Annu. Rev. Med..

[40] Mohanty S., Konkimalla V.B., Pal A., Sharma T., Si S.C. (2021). Naringin as Sustained Delivery Nanoparticles Ameliorates the Anti-inflammatory Activity in a Freund’s Complete Adjuvant-Induced Arthritis Model. ACS Omega.

[41] Ahmed O.M., Ahmed A.A., Fahim H.I., Zaky M.Y. (2019). Quercetin and naringenin abate diethylnitrosamine/acetylaminofluorene-induced hepatocarcinogenesis in Wistar rats: The roles of oxidative stress, inflammation and cell apoptosis. Drug Chem. Toxicol..

[42] Shahin Y.R., Elguindy N.M., Abdel Bary A., Balbaa M. (2018). The protective mechanism of Nigella sativa against diethylnitrosamine-induced hepatocellular carcinoma through its antioxidant effect and EGFR/ERK1/2 signaling. Environ. Toxicol..

[43] Acharya R., Mishra N., Kumar A., Bose P., Pattnaik A., Mukhopadhyay K., Sunita P., Pattanayak S.P. (2021). Naringin, a natural flavonone glycoside attenuates N-nitrosodiethylamine-induced hepatocellular carcinoma in Sprague-Dawley rats. Pharma. Mag..

[44] Ratheesh G., Tian L., Venugopal J.R., Ezhilarasu H., Sadiq A., Fan T.P., Ramakrishna S. (2017). Role of medicinal plants in neurodegenerative diseases. Biomanufactur. Rev..

[45] Park S.J., Jang J.Y., Jeong S.W., Cho Y.K., Lee S.H., Kim S.G., Cha S.W., Kim Y.S., Cho Y.D., Kim H.S. (2017). Usefulness of AFP, AFP-L3, and PIVKA-II, and their combinations in diagnosing hepatocellular carcinoma. Medicine.

[46] Edoo M.I.A., Chutturghoon V.K., Wusu-Ansah G.K., Hai Z.H.U., Zhen T.Y., Xie H.Y., Zheng S.S. (2016). Serum biomarkers AFP, CEA and CA19-9 combined detection for early diagnosis of hepatocellular carcinoma. Iran J. Public Health.

[47] Mansour D.F., Abdallah H.M., Ibrahim B.M., Hegazy R.R., Esmail R.S., Abdel-Salam L.O. (2019). The carcinogenic agent diethylnitrosamine induces early oxidative stress, inflammation and proliferation in rat liver, stomach and colon: Protective effect of ginger extract. Asian Pacific. J. Cancer Prev..

[48] Gunasekaran S., Mayakrishnan V., Al-Ghamdi S., Alsaidan M., Geddawy A., Abdelaziz M.A., Mohideen A.P., Bahakim N.O., Ramesh T., Ayyakannu U.R. (2021). Investigation of phytochemical profile and in vivo anti-proliferative effect of Laetiporus versisporus (Lloyd) Imazeki mushroom against diethylnitrosamine-induced hepatocellular carcinoma. J. King Saud Univ. Sci..

[49] Tao L.Y., Cai L., He X.D., Liu W., Qu Q. (2010). Comparison of serum tumor markers for intrahepatic cholangiocarcinoma and hepatocellular carcinoma. Amer. Sur..

[50] Oliveira M.M., Teixeira J.C., Vasconcelos-Nóbrega C., Felix L.M., Sardão V.A., Colaço A.A., Oliveira P.A., Peixoto F.P. (2013). Mitochondrial and liver oxidative stress alterations induced by N-butyl-N-(4-hydroxybutyl) nitrosamine: Relevance for hepatotoxicity. J. Appl. Toxicol..

[51] Verma A., Singh D., Anwar F., Bhatt P.C., Al-Abbasi F., Kumar V. (2018). Triterpenoids principle of Wedelia calendulacea attenuated diethynitrosamine-induced hepatocellular carcinoma via down-regulating oxidative stress, inflammation and pathology via NF-kB pathway. Inflammopharmacol.

[52] Ahmed O.M. (2016). Relationships between oxidative stress, cancer development and therapeutic interventions. J. Can. Sci. Res..

[53] Yassin N.Y., AbouZid S.F., El-Kalaawy A.M., Ali T.M., Almehmadi M.M., Ahmed O.M. (2022). Silybum marianum total extract, silymarin and silibinin abate hepatocarcinogenesis and hepatocellular carcinoma growth via modulation of the HGF/c-Met, Wnt/β-catenin, and PI3K/Akt/mTOR signaling pathways. Biomed. Pharmacother..

[54] Mo’men Y.S., Hussein R.M., Kandeil M.A. (2020). A novel chemoprotective effect of tiopronin against diethylnitrosamine-induced hepatocellular carcinoma in rats: Role of ASK1/P38 MAPK-P53 signalling cascade. Clin. Exp. Pharmacol. Physiol..

[55] Ribeiro I.A., Rocha J., Sepodes B., Mota-Filipe H., Ribeiro M.H. (2008). Effect of naringin enzymatic hydrolysis towards naringenin on the anti-inflammatory activity of both compounds. J. Mol. Catal. B Enzym..

[56] Thangavel P., Muthu R., Vaiyapuri M. (2012). Antioxidant potential of naringin–A dietary flavonoid–In N-Nitrosodiethylamine induced rat liver carcinogenesis. Biomed. Prev. Nutr..

[57] Adebiyi O.O., Adebiyi O.A., Owira P.M. (2015). Naringin reverses hepatocyte apoptosis and oxidative stress associated with HIV-1 nucleotide reverse transcriptase inhibitors-induced metabolic complications. Nutrients.

[58] Oluwafeyisetan A., Olubunmi A., Peter O. (2016). Naringin ameliorates HIV-1 nucleoside reverse transcriptase inhibitors-induced mitochondrial toxicity. Current HIV Res..

[59] Ali G., Omar H., Hersi F., Abo-Youssef A., Ahmed O., Mohamed W. (2022). The protective role of etoricoxib against diethylnitrosamine/2-acetylaminofluorene-induced hepatocarcinogenesis in Wistar rats: The impact of NF-κB/COX-2/PGE2 signaling. Curr. Mol. Pharmacol..

[60] Zoheir K.M., Abdelhafez M.A., Darwish A.M., Mahrous K.F. (2022). New Approach about the Signaling Crosstalk between IQGAPs/NF-κB/IL-8 and PDCD5/p53/TRAIL Pathways that Modulate Malignant Transformation in Hepatocellular Carcinoma. Asian Pac. J. Cancer Prev..

[61] Uehara T., Pogribny I.P., Rusyn I. (2014). The DEN and CCl4-induced mouse model of fbrosis and infammation-associated hepatocellular carcinoma. Curr. Protoc. Pharmacol..

[62] Singh D., Chaudhary D., Kumar V., Verma A. (2021). Amelioration of diethylnitrosamine (DEN) induced renal oxidative stress and inflammation by Carissa carandas embedded silver nanoparticles in rodents. Toxico. Rep..

[63] Ahmed O.M., Fahim H.I., Ahmed H.Y., Al-Muzafar H.M., Ahmed R.R., Amin K.A., El-Nahass E.S., Abdelazeem W.H. (2019). The preventive effects and the mechanisms of action of navel orange peel hydroethanolic extract, naringin, and naringenin in N-acetyl-p-aminophenol-induced liver injury in Wistar rats. Oxid. Med. Cell. Longev..

[64] Pan H., Wang H., Wang X., Zhu L., Mao L. (2012). The absence of Nrf2 enhances NF-κBdependent inflammation following scratch injury in mouse primary cultured astrocytes. Mediat. Inflamm..

[65] Liu P.L., Tsai J.R., Hwang J.J., Chou S.H., Cheng Y.J., Lin F.Y., Chen Y.L., Hung C.Y., Chen W.C., Chen Y.H. (2010). High-Mobility Group Box 1–Mediated Matrix Metalloproteinase-9 Expression in Non–Small Cell Lung Cancer Contributes to Tumor Cell Invasiveness. Am. J. Resp. Cell Mol. Biol..

[66] Caglayan C., Temel Y., Kandemir F.M., Yildirim S., Kucukler S. (2018). Naringin protects against cyclophosphamide-induced hepatotoxicity and nephrotoxicity through modulation of oxidative stress, inflammation, apoptosis, autophagy, and DNA damage. Environ. Sci. Pollut. Res. Int..

[67] Wu D., Wu P., Zhao L., Huang L., Zhang Z., Zhao S., Huang J. (2015). NF-κB expression and outcomes in solid tumors: A systematic review and meta-analysis. Medicine.

[68] Shirani K., Yousefsani B.S., Shirani M., Karimi G. (2020). Protective effects of naringin against drugs and chemical toxins induced hepatotoxicity: A review. Phytothe. Res..

[69] Lv Z., Wu W., Ge S., Jia R., Lin T., Yuan Y., Kuang H., Yang B., Wu L., Wei J. (2018). Naringin protects against perfluorooctane sulfonate-induced liver injury by modulating NRF2 and NF-κB in mice. Intern. Immunoph..

[70] Dong D., Xu L., Yin L., Qi Y., Peng L. (2015). Naringin prevents carbon tetrachloride-induced acute liver injury in mice. J. Funct. Foods.

[71] Manna K., Khan A., Biswas S., Das U., Sengupta A., Mukherjee D., Chakraborty A., Dey S. (2016). Naringin ameliorates radiation-induced hepatic damage through modulation of Nrf2 and NF-κB pathways. RSC. Adv..

[72] Saraf S. (2010). Applications of novel drug delivery system for herbal formulations. Fitoterapia.

[73] Nair H.B., Sung B., Yadav V.R., Kannappan R., Chaturvedi M.M., Aggarwal B.B. (2010). Delivery of antiinflammatorynutraceuticals by nanoparticles for the prevention and treatment of cancer. Biochem. Pharmacol..

[74] XinYou S., JianQiang Z., Nan L., Kumar M., AiMeiOu Y. (2019). Chemoprotective effects of resveratrol against diethylnitrosamine induced hepatocellular carcinoma in Wistar rats. Inter. J. Pharma..

[75] Medhat A., Mansour S., El-Sonbaty S., Kandil E., Mahmoud M. (2017). Evaluation of the antitumor activity of platinum nanoparticles in the treatment of hepatocellular carcinoma induced in rats. Tumor Biol..

[76] Subramaniam N., Kannan P., Thiruvengadam D. (2019). Hepatoprotective effect of boldine against diethylnitrosamine-induced hepatocarcinogenesis in wistar rats. J. Biochem. Mol. Toxicol..

[77] Kang M.H., Reynolds C.P. (2009). Bcl-2 inhibitors: Targeting mitochondrial apoptotic pathways in cancer therapy. Clin. Cancer Res..

[78] Rückert F., Samm N., Lehner A.K., Saeger H.D., Grützmann R., Pilarsky C. (2020). Simultaneous gene silencing of Bcl-2, XIAP and Survivin re-sensitizes pancreatic cancer cells towards apoptosis. BMC Cancer.

[79] Campbell K.J., Tait S.W. (2018). Targeting BCL-2 regulated apoptosis in cancer. Open Biol..

[80] Marquez R.T., Tsao B.W., Faust N.F., Xu L. (2013). Drug resistance and molecular cancer therapy: Apoptosis versus autophagy. Apoptosis.

[81] Ou X.H., Lu Y., Liao L.F., Li D.N., Liu L.M., Liu H.G., Xu H. (2015). Nitidine chloride induces apoptosis in human hepatocellular carcinoma cells through a pathway involving p53, p21, Bax and Bcl-2. Oncol. Rep..

[82] Cui X., Choi H.K., Choi Y.S., Park S.Y., Sung G.J., Lee Y.H., Lee J., Jun W.J., Kim K., Choi K.C. (2015). DNAJB1 destabilizes PDCD5 to suppress p53-mediated apoptosis. Cancer Lett..

[83] Feng Y., Wang N., Ye X., Li H., Feng Y., Cheung F., Nagamatsu T. (2011). Hepatoprotective effect and its possible mechanism of Coptidis rhizoma aqueous extract on carbon tetrachloride-induced chronic liver hepatotoxicity in rats. J. Ethnopharmacol..

[84] Xie D., Yuan P., Wang D., Jin H., Chen H. (2017). Effects of naringin on the expression of miR-19b and cell apoptosis in human hepatocellular carcinoma. Oncol. Lett..

[85] Zhuge C., Sun X., Chen Y., Lei J. (2016). PDCD5 functions as a regulator of p53 dynamics in the DNA damage response. J. Theoret. Biol..

[86] Borude P., Bhushan B., Chavan H., Weemhoff J.L., Jaeschke H., Krishnamurthy P., Apte U. (2017). P53 regulates progression of injury and liver regeneration after acetaminophen overdose. FASEB J..

[87] Ghanbari-Movahed M., Jackson G., Farzaei M.H., Bishayee A. (2021). A systematic review of the preventive and therapeutic effects of naringin against human malignancies. Front. Pharmacol..

[88] Whittaker S., Marais R., Zhu A.X. (2010). The role of signaling pathways in the development and treatment of hepatocellular carcinoma. Oncogene.

[89] Schmidt V.A. (2012). Watch the GAP: Emerging Roles for IQ Motif-Containing GTPase-Activating Proteins IQGAPs in Hepatocellular Carcinoma. Int. J. Hepatol..

[90] Zoheir K., Abd-Rabou A.A., Harisa G.I., Kumar A., Ahmad S.F., Ansari M.A., Abd-Allah A.R. (2016). IQGAP1 gene silencing induces apoptosis and decreases the invasive capacity of human hepatocellular carcinoma cells. Tumor Biol..

[91] Johnson M., Sharma M., Henderson B.R. (2009). IQGAP1 regulation and roles in cancer. Cell Signal..

[92] White M.J., McArthur K., Metcalf D., Lane R.M., Cambier J.C., Herold M.J., Van Delft M.F., Bedoui S., Lessene G., Ritchie M.E. (2014). Apoptotic caspases suppress mtDNA-induced STING-mediated type I IFN production. Cell.

[93] Gnatenko D.V., Xu X., Zhu W., Schmidt V.A. (2013). Transcript profiling identifies iqgap2−/− mouse as a model for advanced human hepatocellular carcinoma. PLoS ONE.

[94] Skawran B., Steinemann D., Weigmann A., Flemming P., Becker T., Flik J., Kreipe H., Schlegelberger B., Wilkens L. (2008). Gene expression profiling in hepa- tocellular carcinoma: Upregulation of genes in amplified chromosome regions. Mod. Pathol..

[95] Yang Y., Zhao W., Xu Q.W., Wang X.S., Zhang Y., Zhang J. (2014). IQGAP3 promotes EGFR-ERK signaling and the growth and metastasis of lung cancer cells. PLoS ONE.

[96] Qian E.N., Han S.Y., Ding S.Z., Lv X. (2016). Expression and diagnostic value of CCT3 and IQGAP3 in hepatocellular carcinoma. Cancer Cell Inter..

[97] Shi Y., Qin N., Zhou Q., Chen Y., Huang S., Chen B., Shen G., Jia H. (2017). Role of IQGAP3 in metastasis and epithelial–mesenchymal transition in human hepatocellular carcinoma. J. Transl. Med..

[98] Wu J., Chen Z., Cao H., Yu Z., Feng J., Wang K., Lu Q., Wu Y. (2019). High expression of IQGAP3 indicates poor prognosis in colorectal cancer patients. Inter. J. Bio. Markers.

[99] Shahat A.A., Alsaid M.S., Kotob S.E., Ahmed H.H. (2015). Significance of Rumex vesicarius as anticancer remedy against hepatocellular carcinoma: A proposal-based on experimental animal studies. Asian Pac. J. Cancer Prev..

[100] Newell P., Villanueva A., Friedman S.L., Koike K., Llovet J.M. (2008). Experimental models of hepatocellular carcinoma. J. Hepatol..

[101] Dhillon A.S., Hagan S., Rath O., Kolch W. (2007). MAP kinase signalling pathways in cancer. Oncogene.

[102] Castellano E., Downward J. (2011). RAS interaction with PI3K: More than just another effector pathway. Genes Cancer.

[103] Zoheir K.M., Abd-Rabou A.A., Harisa G.I., Ashour A.E., Ahmad S.F., Attia S.M., Bakheet S.A., Abdel-Hamied H.E., Abd-Allah A.R., Kumar A. (2015). Gene expression of IQGAPs and Ras families in an experimental mouse model for hepatocellular carcinoma: A mechanistic study of cancer progression. Int. J. Clin. Exp. Pathol..

[104] Cao Y., Ke R., Wang S., Zhu X., Chen J., Huang C., Jiang Y., Lv L. (2017). EDNA topoisomerase IIα and Ki67 are prognostic factors in patients with hepatocellular carcinoma. Oncol. Lett..

[105] Jagetia G.C., Reddy T.K. (2005). Modulation of radiationinduced alteration in the antioxidant status of mice by naringin. Life Sci..

[106] Kumar A., Prakash A., Dogra S. (2010). Naringin alleviates cognitive impairment, mitochondrial dysfunction and oxidative stress induced by D-galactose in mice. Food Chem. Toxicol..

[107] Thangavel P., Vaiyapuri M. (2013). Antiproliferative and apoptotic effects of naringin on diethylnitrosamine induced hepatocellular carcinoma in rats. Biomed. Aging Pathol..

