# Supplementary material for: Protective Effects of Naringin–Dextrin Nanoformula against Chemically Induced Hepatocellular Carcinoma in Wistar Rats: Roles of Oxidative Stress, Inflammation, Cell Apoptosis, and Proliferation
Source: Pharmaceuticals (Basel). 2022 Dec 14;15(12):1558. doi: 10.3390/ph15121558 (PMC9786090; doi:10.3390/ph15121558)
Supplement: Supplementary file 1 [file pharmaceuticals-15-01558-s001.zip › Supplementary file/Data of Figures.pdf]

Data of Figure 2 : (AFP, CEA and CA19.9)

Group \ Parameter	AFP (ng/ml)	CEA (ng/ml)	CA19.9 (U/L)
**Normal group**	0.63±0.04[a]	2.14±0.05[a]	17.26±0.61[a]
**DEN/2AAF-CG**	3.71±0.25[c]	9.10±0.49[c]	87.23±0.76[c]
**DEN/2AAF-Naringin**	1.63±0.136[b]	3.62±0.25[b]	23.26±1.49[b]
**DEN/2AAF-NDNP**	1.33±0.05[b]	2.86±0.17[ab]	18.71±0.06[a]

Data of Figure 3 (LPO, GSH, GPx and SOD).

Parameter / Group	LPO (nmole MDA /100 mg tissue/hr)	GSH (nmole /100 mg tissue)	GPx (mU/100 mg tissue)	SOD (U/gm tissue)
**Normal group**	16.73±0.42[a]	57.20±0.93[d]	116.79±0.75[c]	28.58±1.11[c]
**DEN/2AAF-CG**	77.70±0.66[c]	22.83±0.40[a]	53.56±0.49[a]	11.01±0.39[a]
**DEN/2AAF-Naringin**	24.43±0.0.27[b]	46.50±0.64[b]	106.08±0.97[b]	22.61±0.48[b]
**DEN/2AAF-NDNP**	17.96±0.23[a]	50.53±2.10[c]	116.16±0.36[c]	28.61±1.23[c]

Data of Figure 4 (A) (NF-κB and IL-8)

Group \ Parameter	NF-κB expressed gene/β actin	IL-8 expressed gene/β actin
**Normal group**	1.00±0.05[a]	1.00±0.05[a]
**DEN/2AAF-CG**	4.6±0.11[d]	2.57±0.25[d]
**DEN/2AA-Naringin**	2.7±0.12[c]	1.42±0.05[c]
**DEN/2AAF-NDNP**	2.3±0.11[b]	1.25±0.04[b]

Data of Figure 4 (B) (Bcl-2, Bax, P53 and PDCD5)

Group \ Parameter	Bcl-2 expressed gene/β-actin	Bax expressed gene/β-actin	P53 expressed gene/β-actin	PDCD5 expressed gene/β-actin
**Normal group**	1.00±0.06[a]	1.00±0.04[a]	1.00±0.04[b]	1.00±0.05[c]
**DEN/2AAF-CG**	2.48±0.25[c]	0.28±0.03[c]	0.54±0.04[a]	0.21±0.05[a]
**DEN/2AAF-Naringin**	1.35±0.11[b]	1.34±0.10[b]	0.94±0.01[b]	0.78±0.02[b]
**DEN/2AAF-NDNP**	1.29±0.11[b]	1.5±0.10[b]	0.97±0.05[b]	0.84±0.02[b]

Data of Figure 4 (C) (IQGAP1, IQGAP2 and IQGAP3)

Parameter / Group	IQGAP1 expressed gene/β-actin	IQGAP2 expressed gene/β-actin	IQGAP3 expressed gene/β-actin
**Normal group**	1.00 ± 0.05^{c}	1.00 ± 0.04^{b}	1.00 ± 0.06^{b}
**DEN/2AAF-CG**	2.98 ± 0.1^{d}	0.55 ± 0.03^{a}	1.8 ± 0.02^{c}
**DEN/2AAF-Naringin**	0.59 ± 0.03^{b}	1.34 ± 0.04^{c}	0.69 ± 0.05^{a}
**DEN/2AAF-NDNP**	0.47 ± 0.02^{a}	1.87 ± 0.02^{d}	0.65 ± 0.04^{a}

Data of Figure 4 (D) (HRAS and KRAS)

parameter / Group	HRAS expressed gene/β-actin	KRAS expressed gene/β-actin
**Normal group**	1.00 ± 0.03^{c}	1.00 ± 0.04^{c}
**DEN/2AAF-CG**	3.08 ± 0.1^{d}	2.95 ± 0.11^{d}
**DEN/2AAF-Naringin**	0.67 ± 0.03^{b}	0.61 ± 0.03^{b}
**DEN/2AAF-NDNP**	0.42 ± 0.02^{a}	0.32 ± 0.01^{a}

Data of Figure 5 (Ki-67)

Group \ Parameter	Ki-67 expressed /β-actin
**Normal group**	1.01±0.046[a]
**DEN/2AAF-CG**	3.11±0.67[d]
**DEN/2AAF-Naringin**	1.69±0.043[c]
**DEN/2AAF-NDNP**	1.25±0.025[b]
