# Supplementary material for: Protective Effects of Naringin–Dextrin Nanoformula against Chemically Induced Hepatocellular Carcinoma in Wistar Rats: Roles of Oxidative Stress, Inflammation, Cell Apoptosis, and Proliferation
Source: Pharmaceuticals (Basel). 2022 Dec 14;15(12):1558. doi: 10.3390/ph15121558 (PMC9786090; doi:10.3390/ph15121558)
Supplement: Supplementary file 1 [file pharmaceuticals-15-01558-s001.zip › Supplementary file/Statistics outputs - Liver parameters.pdf]

```
MEANS TABLES=alt BY alt1
  /CELLS MEAN SEMEAN.
```

Means

Notes

Output Created		07-JUN-2021 09:04:11
Comments		
Input	Data	D:\Eman\الدكتوراه\PhD\PhD الكتابه\نتايج الاحصاء\New folder\alt.sav
	Active Dataset	DataSet1
	Filter	<none>
	Weight	<none>
	Split File	<none>
	N of Rows in Working Data File	24
Missing Value Handling	Definition of Missing	For each dependent variable in a table, user-defined missing values for the dependent and all grouping variables are treated as missing.
	Cases Used	Cases used for each table have no missing values in any independent variable, and not all dependent variables have missing values.
Syntax		MEANS TABLES=alt BY alt1 /CELLS MEAN SEMEAN.
Resources	Processor Time	00:00:00.00
	Elapsed Time	00:00:00.00

```
[DataSet1] D:\Eman\الدكتوراه\PhD\PhD الاحصاء نتايج\الكتابه\New folder\alt.sav
```

Case Processing Summary

	Cases					
	Included		Excluded		Total	
	N	Percent	N	Percent	N	Percent
alt * alt1	24	100.0%	0	0.0%	24	100.0%

Report

alt

alt1	Mean	Std. Error of Mean
1.00	40.4000	3.05287
2.00	70.1833	2.68731
3.00	50.3167	1.31945
4.00	41.3667	1.73141
Total	50.5667	2.71725

```
ONEWAY alt BY alt1
  /MISSING ANALYSIS
  /POSTHOC=DUNCAN ALPHA(0.05).
```

Oneway

Notes

Output Created		07-JUN-2021 09:04:27
Comments		
Input	Data	D:\Eman\الدكتوراه\PhD\PhD الاحصاء نتايج\الكتابه\New folder\alt.sav
	Active Dataset	DataSet1

	Filter	<none>
	Weight	<none>
	Split File	<none>
	N of Rows in Working Data File	24
Missing Value Handling	Definition of Missing	User-defined missing values are treated as missing.
	Cases Used	Statistics for each analysis are based on cases with no missing data for any variable in the analysis.
Syntax		ONEWAY alt BY alt1<br>/MISSING ANALYSIS<br>/POSTHOC=DUNCAN ALPHA(0.05).
Resources	Processor Time	00:00:00.02
	Elapsed Time	00:00:00.03

[DataSet1] D:\Eman\الدكتوراه\PhD\PhD نتايج الاحصاء\الكتابه\New folder\alt.sav

ANOVA

alt

	Sum of Squares	df	Mean Square	F	Sig.
Between Groups	3437.263	3	1145.754	35.894	.000
Within Groups	638.410	20	31.920		
Total	4075.673	23			

Post Hoc Tests

Homogeneous Subsets

alt

Duncan

alt1	N	Subset for alpha = 0.05		
		1	2	3
1.00	6	40.4000		
4.00	6	41.3667		
3.00	6		50.3167	
2.00	6			70.1833
Sig.		.770	1.000	1.000

Means for groups in homogeneous subsets are displayed.

a. Uses Harmonic Mean Sample Size = 6.000.

```
GET
  FILE='D:\Eman\_75;ه;75_;85_و;78_ك;83_ل\PhD\PhD
_75;69_;75_;89_;81_;75_ل;75_ ;80_ي;75_;78_ن\ه;76_;75_;78_لك;\New
folder\AST.sav'.
DATASET NAME DataSet1 WINDOW=FRONT.
MEANS TABLES=AST BY AST1
  /CELLS MEAN SEMEAN.
```

Means

Notes

Output Created	07-JUN-2021 09:13:38
Comments	

Input	Data	D:\Eman\الدكتوراه\PhD\PhD الكتابه\نتايج الاحصاء\New folder\AST.sav
	Active Dataset	DataSet1
	Filter	<none>
	Weight	<none>
	Split File	<none>
	N of Rows in Working Data File	24
Missing Value Handling	Definition of Missing	For each dependent variable in a table, user-defined missing values for the dependent and all grouping variables are treated as missing.
	Cases Used	Cases used for each table have no missing values in any independent variable, and not all dependent variables have missing values.
Syntax		MEANS TABLES=AST BY AST1<br>/CELLS MEAN SEMEAN.
Resources	Processor Time	00:00:00.02
	Elapsed Time	00:00:00.02

```
[DataSet1] D:\Eman\الدكتوراه\PhD\PhD الاحصاء نتايج\الكتابه\New folder\AST.sav
```

Case Processing Summary

	Cases					
	Included		Excluded		Total	
	N	Percent	N	Percent	N	Percent
AST * AST1	24	100.0%	0	0.0%	24	100.0%

Report

AST

AST1	Mean	Std. Error of Mean
1.00	97.0000	1.52753
2.00	161.6667	2.96273
3.00	111.1667	4.24591
4.00	100.5000	2.51330
Total	117.5833	5.59241

```
ONEWAY AST BY AST1
  /MISSING ANALYSIS
  /POSTHOC=DUNCAN ALPHA(0.05).
```

Oneway

Notes

Output Created		07-JUN-2021 09:14:04
Comments		
Input	Data	D:\Eman\الدكتوراه\PhD\PhD الكتابه\نتايج الاحصاء\New folder\AST.sav
	Active Dataset	DataSet1
	Filter	<none>
	Weight	<none>
	Split File	<none>
	N of Rows in Working Data File	24
Missing Value Handling	Definition of Missing	User-defined missing values are treated as missing.
	Cases Used	Statistics for each analysis are based on cases with no missing data for any variable in the analysis.

Syntax		ONEWAY AST BY AST1<br>/MISSING ANALYSIS<br>/POSTHOC=DUNCAN ALPHA(0.05).
Resources	Processor Time	00:00:00.02
	Elapsed Time	00:00:00.02

```
[DataSet1] D:\Eman\الدكتوراه\PhD\PhD الاحصاء نتايج\الكتابه\New folder\AST.sav
```

ANOVA

AST

	Sum of Squares	df	Mean Square	F	Sig.
Between Groups	16200.167	3	5400.056	101.537	.000
Within Groups	1063.667	20	53.183		
Total	17263.833	23			

Post Hoc Tests

Homogeneous Subsets

AST

Duncan

AST1	N	Subset for alpha = 0.05		
		1	2	3
1.00	6	97.0000		
4.00	6	100.5000		
3.00	6		111.1667	
2.00	6			161.6667

Sig.		.416	1.000	1.000

Means for groups in homogeneous subsets are displayed.

a. Uses Harmonic Mean Sample Size = 6.000.

```
MEANS TABLES=ALP BY ALP1
  /CELLS MEAN SEMEAN.
```

Means

Notes

Output Created		07-JUN-2021 08:33:23
Comments		
Input	Data	D:\Eman\الدكتوراه\PhD\PhD الكتابه\نتايج الاحصاء\New folder\ALP.sav
	Active Dataset	DataSet1
	Filter	<none>
	Weight	<none>
	Split File	<none>
	N of Rows in Working Data File	24
Missing Value Handling	Definition of Missing	For each dependent variable in a table, user-defined missing values for the dependent and all grouping variables are treated as missing.
	Cases Used	Cases used for each table have no missing values in any independent variable, and not all dependent variables have missing values.

Syntax		MEANS TABLES=ALP BY ALP1<br>/CELLS MEAN SEMEAN.
Resources	Processor Time	00:00:00.00
	Elapsed Time	00:00:00.00

```
[DataSet1] D:\Eman\الدكتوراه\PhD\PhD الاحصاء نتايج\الكتابه\New folder\ALP.sav
```

Case Processing Summary

	Cases					
	Included		Excluded		Total	
	N	Percent	N	Percent	N	Percent
ALP * ALP1	24	100.0%	0	0.0%	24	100.0%

Report

ALP

ALP1	Mean	Std. Error of Mean
1.00	206.0833	6.45809
2.00	506.2833	11.46300
3.00	398.6667	10.59769
4.00	338.6667	10.69787
Total	362.4250	23.08189

```
ONEWAY ALP BY ALP1
  /MISSING ANALYSIS
  /POSTHOC=DUNCAN ALPHA(0.05).
```

Oneway

Notes

Output Created		07-JUN-2021 08:33:39
Comments		
Input	Data	D:\Eman\الدكتوراه\PhD\PhD الكتابه\نتايج الاحصاء\New folder\ALP.sav
	Active Dataset	DataSet1
	Filter	<none>
	Weight	<none>
	Split File	<none>
	N of Rows in Working Data File	24
Missing Value Handling	Definition of Missing	User-defined missing values are treated as missing.
	Cases Used	Statistics for each analysis are based on cases with no missing data for any variable in the analysis.
Syntax		ONEWAY ALP BY ALP1<br>/MISSING ANALYSIS<br>/POSTHOC=DUNCAN ALPHA(0.05).
Resources	Processor Time	00:00:00.03
	Elapsed Time	00:00:00.03

```
[DataSet1] D:\Eman\الدكتوراه\PhD\PhD الكتابه\نتايج الاحصاء\New folder\ALP.sav
```

ANOVA

ALP

	Sum of Squares	df	Mean Square	F	Sig.
Between Groups	282095.122	3	94031.707	156.773	.000
Within Groups	11995.883	20	599.794		
Total	294091.005	23			

Post Hoc Tests

Homogeneous Subsets

ALP

Duncan

ALP1	N	Subset for alpha = 0.05			
		1	2	3	4
1.00	6	206.0833			
4.00	6		338.6667		
3.00	6			398.6667	
2.00	6				506.2833
Sig.		1.000	1.000	1.000	1.000

Means for groups in homogeneous subsets are displayed.

a. Uses Harmonic Mean Sample Size = 6.000.

```
MEANS TABLES=albumin BY albumin1
  /CELLS MEAN SEMEAN.
```

Means

Notes

Output Created	07-JUN-2021 08:54:40
Comments	

Input	Data	D:\Eman\الدكتوراه\PhD\PhD الكتابه\نتايج الاحصاء\New folder\albumin.sav
	Active Dataset	DataSet1
	Filter	<none>
	Weight	<none>
	Split File	<none>
	N of Rows in Working Data File	24
Missing Value Handling	Definition of Missing	For each dependent variable in a table, user-defined missing values for the dependent and all grouping variables are treated as missing.
	Cases Used	Cases used for each table have no missing values in any independent variable, and not all dependent variables have missing values.
Syntax		MEANS TABLES=albumin BY albumin1 /CELLS MEAN SEMEAN.
Resources	Processor Time	00:00:00.00
	Elapsed Time	00:00:00.00

[DataSet1] D:\Eman\الدكتوراه\PhD\PhD الكتابه\نتايج الاحصاء\New folder\albumin.sav

Case Processing Summary

	Cases					
	Included		Excluded		Total	
	N	Percent	N	Percent	N	Percent
albumin * albumin1	24	100.0%	0	0.0%	24	100.0%

Report

albumin

albumin1	Mean	Std. Error of Mean
1.00	3.7550	.11687
2.00	2.8817	.05546
3.00	3.3233	.07102
4.00	3.5533	.04216
Total	3.3783	.07661

```
ONEWAY albumin BY albumin1
  /MISSING ANALYSIS
  /POSTHOC=DUNCAN ALPHA(0.05).
```

Oneway

Notes

Output Created		07-JUN-2021 08:54:53
Comments		
Input	Data	D:\Eman\الدكتوراه\PhD\PhD الكتابه\نتايج الاحصاء\New folder\albumin.sav
	Active Dataset	DataSet1
	Filter	<none>
	Weight	<none>
	Split File	<none>
	N of Rows in Working Data File	24
Missing Value Handling	Definition of Missing	User-defined missing values are treated as missing.
	Cases Used	Statistics for each analysis are based on cases with no missing data for any variable in the analysis.

Syntax		ONEWAY albumin BY albumin1<br>/MISSING ANALYSIS<br>/POSTHOC=DUNCAN ALPHA(0.05).
Resources	Processor Time	00:00:00.02
	Elapsed Time	00:00:00.05

[DataSet1] D:\Eman\الدكتوراه\PhD\PhD نتايج الاحصاء\الكتابه\New folder\albumin.sav

ANOVA

albumin

	Sum of Squares	df	Mean Square	F	Sig.
Between Groups	2.533	3	.844	23.897	.000
Within Groups	.707	20	.035		
Total	3.240	23			

Post Hoc Tests

Homogeneous Subsets

albumin

Duncan

albumin1	N	Subset for alpha = 0.05		
		1	2	3
2.00	6	2.8817		
3.00	6		3.3233	
4.00	6			3.5533

1.00	6			3.7550
Sig.		1.000	1.000	.078

Means for groups in homogeneous subsets are displayed.

a. Uses Harmonic Mean Sample Size = 6.000.

```
GET
  FILE='D:\Eman\_75; ;80_ي;75_;78_ن\ه;75_;85_و;78_ك;83_ل
69_;75_;89_;81_;75_ل;75_;\New folder\bilirubin.sav'.
DATASET NAME DataSet1 WINDOW=FRONT.
MEANS TABLES=bilirubin BY bilirubin1
  /CELLS MEAN SEMEAN.
```

Means

Notes

Output Created		24-FEB-2021 05:41:13
Comments		
Input	Data	D:\Eman\ الدكتوراه\نتايج الاحصاء\New folder\bilirubin.sav
	Active Dataset	DataSet1
	Filter	<none>
	Weight	<none>
	Split File	<none>
	N of Rows in Working Data File	24

Missing Value Handling	Definition of Missing	For each dependent variable in a table, user-defined missing values for the dependent and all grouping variables are treated as missing.
	Cases Used	Cases used for each table have no missing values in any independent variable, and not all dependent variables have missing values.
Syntax		MEANS TABLES=bilirubin BY bilirubin1 /CELLS MEAN SEMEAN.
Resources	Processor Time	00:00:00.02
	Elapsed Time	00:00:00.01

[DataSet1] D:\Eman\نتايج الاحصاء\الدكتوراه\New folder\bilirubin.sav

Case Processing Summary

	Cases					
	Included		Excluded		Total	
	N	Percent	N	Percent	N	Percent
bilirubin * bilirubin1	24	100.0%	0	0.0%	24	100.0%

Report

bilirubin

bilirubin1	Mean	Std. Error of Mean
1.00	.2767	.02140
2.00	.9017	.07926
3.00	.4800	.03821
4.00	.4050	.04048

Total	.5158	.05406

```
ONEWAY bilirubin BY bilirubin1
  /MISSING ANALYSIS
  /POSTHOC=DUNCAN ALPHA(0.05).
```

Oneway

Notes

Output Created		24-FEB-2021 05:41:34
Comments		
Input	Data	D:\Eman\ الدكتوراه\نتايج الاحصاء\New folder\bilirubin.sav
	Active Dataset	DataSet1
	Filter	<none>
	Weight	<none>
	Split File	<none>
	N of Rows in Working Data File	24
Missing Value Handling	Definition of Missing	User-defined missing values are treated as missing.
	Cases Used	Statistics for each analysis are based on cases with no missing data for any variable in the analysis.
Syntax		ONEWAY bilirubin BY bilirubin1 /MISSING ANALYSIS /POSTHOC=DUNCAN ALPHA(0.05).
Resources	Processor Time	00:00:00.02
	Elapsed Time	00:00:00.03

```
[DataSet1] D:\Eman\الدكتوراه\نتايج الاحصاء\New folder\bilirubin.sav
```

ANOVA

bilirubin

	Sum of Squares	df	Mean Square	F	Sig.
Between Groups	1.318	3	.439	29.764	.000
Within Groups	.295	20	.015		
Total	1.613	23			

Post Hoc Tests

Homogeneous Subsets

bilirubin

Duncan

bilirubin1	N	Subset for alpha = 0.05		
		1	2	3
1.00	6	.2767		
4.00	6	.4050	.4050	
3.00	6		.4800	
2.00	6			.9017
Sig.		.082	.298	1.000

Means for groups in homogeneous subsets are displayed.

a. Uses Harmonic Mean Sample Size = 6.000.

```
MEANS TABLES=AFP BY AFP1
  /CELLS MEAN SEMEAN.
```

Means

Notes

Output Created		14-FEB-2021 08:29:39
Comments		
Input	Active Dataset	DataSet0
	Filter	<none>
	Weight	<none>
	Split File	<none>
	N of Rows in Working Data File	24
Missing Value Handling	Definition of Missing	For each dependent variable in a table, user-defined missing values for the dependent and all grouping variables are treated as missing.
	Cases Used	Cases used for each table have no missing values in any independent variable, and not all dependent variables have missing values.
Syntax		MEANS TABLES=AFP BY AFP1 /CELLS MEAN SEMEAN.
Resources	Processor Time	00:00:00.00
	Elapsed Time	00:00:00.00

[DataSet0]

Case Processing Summary

	Cases					
	Included		Excluded		Total	
	N	Percent	N	Percent	N	Percent
AFP * AFP1	24	100.0%	0	0.0%	24	100.0%

Report

AFP

AFP1	Mean	Std. Error of Mean
1.00	.6333	.04766
2.00	3.7167	.25970
3.00	1.6333	.13824
4.00	1.3333	.05578
Total	1.8292	.24971

```
ONEWAY AFP BY AFP1
  /MISSING ANALYSIS
  /POSTHOC=DUNCAN ALPHA(0.05).
```

Oneway

Notes

Output Created		14-FEB-2021 08:30:03
Comments		
Input	Active Dataset	DataSet0
	Filter	<none>
	Weight	<none>
	Split File	<none>
	N of Rows in Working Data File	24
Missing Value Handling	Definition of Missing	User-defined missing values are treated as missing.

	Cases Used	Statistics for each analysis are based on cases with no missing data for any variable in the analysis.
Syntax		ONEWAY AFP BY AFP1<br>/MISSING ANALYSIS<br>/POSTHOC=DUNCAN ALPHA(0.05).
Resources	Processor Time	00:00:00.02
	Elapsed Time	00:00:00.02

[DataSet0]

ANOVA

AFP

	Sum of Squares	df	Mean Square	F	Sig.
Between Groups	31.661	3	10.554	76.528	.000
Within Groups	2.758	20	.138		
Total	34.419	23			

Post Hoc Tests

Homogeneous Subsets

AFP

Duncan

AFP1	N	Subset for alpha = 0.05		
		1	2	3

1.00	6	.6333		
4.00	6		1.3333	
3.00	6		1.6333	
2.00	6			3.7167
Sig.		1.000	.177	1.000

Means for groups in homogeneous subsets are displayed.

a. Uses Harmonic Mean Sample Size = 6.000.

```
MEANS TABLES=CEA BY CEA1
  /CELLS MEAN SEMEAN.
```

Means

Notes

Output Created		14-FEB-2021 08:52:43
Comments		
Input	Active Dataset	DataSet0
	Filter	<none>
	Weight	<none>
	Split File	<none>
	N of Rows in Working Data File	24
Missing Value Handling	Definition of Missing	For each dependent variable in a table, user-defined missing values for the dependent and all grouping variables are treated as missing.
	Cases Used	Cases used for each table have no missing values in any independent variable, and not all dependent variables have missing values.

Syntax		MEANS TABLES=CEA BY CEA1<br>/CELLS MEAN SEMEAN.
Resources	Processor Time	00:00:00.00
	Elapsed Time	00:00:00.00

```
[DataSet0]
```

Case Processing Summary

	Cases					
	Included		Excluded		Total	
	N	Percent	N	Percent	N	Percent
CEA * CEA1	24	100.0%	0	0.0%	24	100.0%

Report

CEA

CEA1	Mean	Std. Error of Mean
1.00	2.1400	.05266
2.00	9.1000	.49126
3.00	3.6200	.25941
4.00	2.8667	.17256
Total	4.4317	.58847

```
ONEWAY CEA BY CEA1
  /MISSING ANALYSIS
  /POSTHOC=DUNCAN ALPHA(0.05).
```

Oneway

Notes

Output Created		14-FEB-2021 08:53:07
Comments		
Input	Active Dataset	DataSet0
	Filter	<none>
	Weight	<none>
	Split File	<none>
	N of Rows in Working Data File	24
Missing Value Handling	Definition of Missing	User-defined missing values are treated as missing.
	Cases Used	Statistics for each analysis are based on cases with no missing data for any variable in the analysis.
Syntax		ONEWAY CEA BY CEA1 /MISSING ANALYSIS /POSTHOC=DUNCAN ALPHA(0.05).
Resources	Processor Time	00:00:00.02
	Elapsed Time	00:00:00.06

[DataSet0]

ANOVA

CEA

	Sum of Squares	df	Mean Square	F	Sig.
Between Groups	180.919	3	60.306	117.839	.000
Within Groups	10.235	20	.512		
Total	191.154	23			

Post Hoc Tests

Homogeneous Subsets

CEA

Duncan

CEA1	N	Subset for alpha = 0.05		
		1	2	3
1.00	6	2.1400		
4.00	6	2.8667	2.8667	
3.00	6		3.6200	
2.00	6			9.1000
Sig.		.094	.083	1.000

Means for groups in homogeneous subsets are displayed.

a. Uses Harmonic Mean Sample Size = 6.000.

```
GET
  FILE='D:\69_;75_;89_;81_;75_ل;75_ ;80_ي;75_;78_ن;\New folder\CA.sav'.
DATASET NAME DataSet1 WINDOW=FRONT.
MEANS TABLES=CA BY CA1
  /CELLS MEAN SEMEAN.
```

Means

Notes

Output Created		15-FEB-2021 23:07:58
Comments		
Input	Data	D:\نتايج الاحصاء\New folder\CA.sav
	Active Dataset	DataSet1
	Filter	<none>

	Weight	<none>
	Split File	<none>
	N of Rows in Working Data File	24
Missing Value Handling	Definition of Missing	For each dependent variable in a table, user-defined missing values for the dependent and all grouping variables are treated as missing.
	Cases Used	Cases used for each table have no missing values in any independent variable, and not all dependent variables have missing values.
Syntax		MEANS TABLES=CA BY CA1 /CELLS MEAN SEMEAN.
Resources	Processor Time	00:00:00.02
	Elapsed Time	00:00:00.02

[DataSet1] D:\الاحصاء نتايج\New folder\CA.sav

Case Processing Summary

	Cases					
	Included		Excluded		Total	
	N	Percent	N	Percent	N	Percent
CA * CA1	24	100.0%	0	0.0%	24	100.0%

Report

CA

CA1	Mean	Std. Error of Mean
1.00	17.2667	.61788

2.00	87.2333	.76797
3.00	23.2667	1.49948
4.00	18.7500	.06708
Total	36.6292	6.12375

```
ONEWAY CA BY CA1
  /MISSING ANALYSIS
  /POSTHOC=DUNCAN ALPHA(0.05).
```

Oneway

Notes

Output Created		15-FEB-2021 23:08:53
Comments		
Input	Data	D:\نتايج الاحصاء\New folder\CA.sav
	Active Dataset	DataSet1
	Filter	<none>
	Weight	<none>
	Split File	<none>
	N of Rows in Working Data File	24
Missing Value Handling	Definition of Missing	User-defined missing values are treated as missing.
	Cases Used	Statistics for each analysis are based on cases with no missing data for any variable in the analysis.
Syntax		ONEWAY CA BY CA1 /MISSING ANALYSIS /POSTHOC=DUNCAN ALPHA(0.05).
Resources	Processor Time	00:00:00.03
	Elapsed Time	00:00:00.03

[DataSet1] D:\نتايج الاحصاء\New folder\CA.sav

ANOVA

CA

	Sum of Squares	df	Mean Square	F	Sig.
Between Groups	20603.455	3	6867.818	1419.924	.000
Within Groups	96.735	20	4.837		
Total	20700.190	23			

Post Hoc Tests

Homogeneous Subsets

CA

Duncan

CA1	N	Subset for alpha = 0.05		
		1	2	3
1.00	6	17.2667		
4.00	6	18.7500		
3.00	6		23.2667	
2.00	6			87.2333
Sig.		.256	1.000	1.000

Means for groups in homogeneous subsets are displayed.

a. Uses Harmonic Mean Sample Size = 6.000.

```
MEANS TABLES=ILB.LIVER BY ILB.LIVER1
  /CELLS MEAN SEMEAN.
```

Means

Notes

Output Created		15-FEB-2021 13:29:14
Comments		
Input	Active Dataset	DataSet0
	Filter	<none>
	Weight	<none>
	Split File	<none>
	N of Rows in Working Data File	24
Missing Value Handling	Definition of Missing	For each dependent variable in a table, user-defined missing values for the dependent and all grouping variables are treated as missing.
	Cases Used	Cases used for each table have no missing values in any independent variable, and not all dependent variables have missing values.
Syntax		MEANS TABLES=ILB.LIVER BY ILB.LIVER1 /CELLS MEAN SEMEAN.
Resources	Processor Time	00:00:00.00
	Elapsed Time	00:00:00.00

```
[DataSet0]
```

Case Processing Summary

	Cases					
	Included		Excluded		Total	
	N	Percent	N	Percent	N	Percent
ILB.LIVER * ILB.LIVER1	24	100.0%	0	0.0%	24	100.0%

Report

ILB.LIVER

ILB.LIVER1	Mean	Std. Error of Mean
1.00	35.4667	1.07228
2.00	165.7667	.80526
3.00	74.1333	.74863
4.00	55.4167	.59353
Total	82.6958	10.40609

```
ONEWAY ILB.LIVER BY ILB.LIVER1
  /MISSING ANALYSIS
  /POSTHOC=DUNCAN ALPHA(0.05).
```

Oneway

Notes

Output Created		15-FEB-2021 13:29:38
Comments		
Input	Active Dataset	DataSet0
	Filter	<none>
	Weight	<none>
	Split File	<none>

	N of Rows in Working Data File	24
Missing Value Handling	Definition of Missing	User-defined missing values are treated as missing.
	Cases Used	Statistics for each analysis are based on cases with no missing data for any variable in the analysis.
Syntax		ONEWAY ILB.LIVER BY ILB.LIVER1 /MISSING ANALYSIS /POSTHOC=DUNCAN ALPHA(0.05).
Resources	Processor Time	00:00:00.00
	Elapsed Time	00:00:00.02

[DataSet0]

ANOVA

ILB.LIVER

	Sum of Squares	df	Mean Square	F	Sig.
Between Groups	59692.961	3	19897.654	4893.166	.000
Within Groups	81.328	20	4.066		
Total	59774.290	23			

Post Hoc Tests

Homogeneous Subsets

ILB.LIVER

Duncan

ILB.LIVER1	N	Subset for alpha = 0.05			
		1	2	3	4
1.00	6	35.4667			
4.00	6		55.4167		
3.00	6			74.1333	
2.00	6				165.7667
Sig.		1.000	1.000	1.000	1.000

Means for groups in homogeneous subsets are displayed.

a. Uses Harmonic Mean Sample Size = 6.000.

```
GET
  FILE='D:\69_;75_;89_;81_;75_ل;75_ ;80_ي;75_;78_ن;\New folder\tnf.sav'.
DATASET NAME DataSet1 WINDOW=FRONT.
MEANS TABLES=tnf.liver BY tnf.liver1
  /CELLS MEAN SEMEAN.
```

Means

Notes

Output Created		15-FEB-2021 13:11:15
Comments		
Input	Data	D:\نتايج الاحصاء\New folder\tnf.sav
	Active Dataset	DataSet1
	Filter	<none>
	Weight	<none>
	Split File	<none>
	N of Rows in Working Data File	24

Missing Value Handling	Definition of Missing	For each dependent variable in a table, user-defined missing values for the dependent and all grouping variables are treated as missing.
	Cases Used	Cases used for each table have no missing values in any independent variable, and not all dependent variables have missing values.
Syntax		MEANS TABLES=tnf.liver BY tnf.liver1 /CELLS MEAN SEMEAN.
Resources	Processor Time	00:00:00.02
	Elapsed Time	00:00:00.02

[DataSet1] D:\الاحصاء نتايج\New folder\tnf.sav

Case Processing Summary

	Cases					
	Included		Excluded		Total	
	N	Percent	N	Percent	N	Percent
tnf.liver * tnf.liver1	24	100.0%	0	0.0%	24	100.0%

Report

tnf.liver

tnf.liver1	Mean	Std. Error of Mean
1.00	22.5333	1.04839
2.00	126.6667	.93903
3.00	55.2333	.78514
4.00	34.0000	.77889

Total	59.6083	8.44569

```
ONEWAY tnf.liver BY tnf.liver1
  /MISSING ANALYSIS
  /POSTHOC=DUNCAN ALPHA(0.05).
```

Oneway

Notes

Output Created		15-FEB-2021 13:12:03
Comments		
Input	Data	D:\نتايج الاحصاء\New folder\tnf.sav
	Active Dataset	DataSet1
	Filter	<none>
	Weight	<none>
	Split File	<none>
	N of Rows in Working Data File	24
Missing Value Handling	Definition of Missing	User-defined missing values are treated as missing.
	Cases Used	Statistics for each analysis are based on cases with no missing data for any variable in the analysis.
Syntax		ONEWAY tnf.liver BY tnf.liver1 /MISSING ANALYSIS /POSTHOC=DUNCAN ALPHA(0.05).
Resources	Processor Time	00:00:00.02
	Elapsed Time	00:00:00.05

```
[DataSet1] D:\نتايج الاحصاء\New folder\tnf.sav
```

ANOVA

tnf.liver

	Sum of Squares	df	Mean Square	F	Sig.
Between Groups	39277.818	3	13092.606	2724.221	.000
Within Groups	96.120	20	4.806		
Total	39373.938	23			

Post Hoc Tests

Homogeneous Subsets

tnf.liver

Duncan

tnf.liver1	N	Subset for alpha = 0.05			
		1	2	3	4
1.00	6	22.5333			
4.00	6		34.0000		
3.00	6			55.2333	
2.00	6				126.6667
Sig.		1.000	1.000	1.000	1.000

Means for groups in homogeneous subsets are displayed.

a. Uses Harmonic Mean Sample Size = 6.000.

```
GET
  FILE='D:\Eman\_75; ;80_ي;75_;78_ن\ه;75_;85_و;78_ك;83_ل
69_;75_;89_;81_;75_ل;75_;\New folder\NRF2.LIVER.sav'.
DATASET NAME DataSet2 WINDOW=FRONT.
MEANS TABLES=NRF2.LIVER BY NRF2.LIVER1
  /CELLS MEAN SEMEAN.
```

Means

Notes

Output Created		23-FEB-2021 17:01:36
Comments		
Input	Data	D:\Eman\الدكتوراه\نتايج الاحصاء\New folder\NRF2.LIVER.sav
	Active Dataset	DataSet2
	Filter	<none>
	Weight	<none>
	Split File	<none>
	N of Rows in Working Data File	24
Missing Value Handling	Definition of Missing	For each dependent variable in a table, user-defined missing values for the dependent and all grouping variables are treated as missing.
	Cases Used	Cases used for each table have no missing values in any independent variable, and not all dependent variables have missing values.

Syntax		MEANS TABLES=NRF2.LIVER BY NRF2.LIVER1 /CELLS MEAN SEMEAN.
Resources	Processor Time	00:00:00.00
	Elapsed Time	00:00:00.01

```
[DataSet2] D:\Eman\الاحصاء نتايج\الدكتوراه\New folder\NRF2.LIVER.sav
```

Case Processing Summary

	Cases					
	Included		Excluded		Total	
	N	Percent	N	Percent	N	Percent
NRF2.LIVER * NRF2.LIVER1	24	100.0%	0	0.0%	24	100.0%

Report

NRF2.LIVER

NRF2.LIVER1	Mean	Std. Error of Mean
1.00	259.4000	2.81591
2.00	93.8000	.77503
3.00	219.4167	1.21337
4.00	236.5167	3.12649
Total	202.2833	13.43070

```
ONEWAY NRF2.LIVER BY NRF2.LIVER1
  /MISSING ANALYSIS
  /POSTHOC=DUNCAN ALPHA(0.05).
```

Oneway

Notes

Output Created		23-FEB-2021 17:02:24
Comments		
Input	Data	D:\Eman\ الدكتوراه\نتايج الاحصاء\New folder\NRF2.LIVER.sav
	Active Dataset	DataSet2
	Filter	<none>
	Weight	<none>
	Split File	<none>
	N of Rows in Working Data File	24
Missing Value Handling	Definition of Missing	User-defined missing values are treated as missing.
	Cases Used	Statistics for each analysis are based on cases with no missing data for any variable in the analysis.
Syntax		ONEWAY NRF2.LIVER BY NRF2.LIVER1 /MISSING ANALYSIS /POSTHOC=DUNCAN ALPHA(0.05).
Resources	Processor Time	00:00:00.03
	Elapsed Time	00:00:00.04

[DataSet2] D:\Eman\الدكتوراه\نتايج الاحصاء\New folder\NRF2.LIVER.sav

ANOVA

NRF2.LIVER

	Sum of Squares	df	Mean Square	F	Sig.
Between Groups	98978.517	3	32992.839	1112.149	.000
Within Groups	593.317	20	29.666		
Total	99571.833	23			

Post Hoc Tests

Homogeneous Subsets

NRF2.LIVER

Duncan

NRF2.LIVER1	N	Subset for alpha = 0.05			
		1	2	3	4
2.00	6	93.8000			
3.00	6		219.4167		
4.00	6			236.5167	
1.00	6				259.4000
Sig.		1.000	1.000	1.000	1.000

Means for groups in homogeneous subsets are displayed.

a. Uses Harmonic Mean Sample Size = 6.000.

```
GET
  FILE='D:\Eman\_75;ه;75_;85_و;78_ك;83_ل\PhD\PhD
_75;69_;75_;89_;81_;75_ل;75_ ;80_ي;75_;78_ن\ه;76_;75_;78_لـك;\New
folder\SOD.LIVER.sav'.
DATASET NAME DataSet1 WINDOW=FRONT.
MEANS TABLES=SOD.LIVER BY SOD.LIVER1
  /CELLS MEAN SEMEAN.
```

Means

Notes

Output Created		15-JUN-2021 05:38:18
Comments		
Input	Data	D:\Eman\الدكتوراه\PhD\PhD الكتابه\نتايج الاحصاء\New folder\SOD.LIVER.sav
	Active Dataset	DataSet1
	Filter	<none>
	Weight	<none>
	Split File	<none>
	N of Rows in Working Data File	24
Missing Value Handling	Definition of Missing	For each dependent variable in a table, user-defined missing values for the dependent and all grouping variables are treated as missing.
	Cases Used	Cases used for each table have no missing values in any independent variable, and not all dependent variables have missing values.
Syntax		MEANS TABLES=SOD.LIVER BY SOD.LIVER1 /CELLS MEAN SEMEAN.
Resources	Processor Time	00:00:00.02
	Elapsed Time	00:00:00.01

[DataSet1] D:\Eman\الدكتوراه\PhD\PhD الكتابه\نتايج الاحصاء\New folder\SOD.LIVER.sav

Case Processing Summary

	Cases

	Included		Excluded		Total	
	N	Percent	N	Percent	N	Percent
SOD.LIVER * SOD.LIVER1	24	100.0%	0	0.0%	24	100.0%

Report

SOD.LIVER

SOD.LIVER1	Mean	Std. Error of Mean
1.00	28.5833	1.11308
2.00	11.0167	.39700
3.00	22.6167	.48677
4.00	28.6167	1.23273
Total	22.7083	1.55302

```
ONEWAY SOD.LIVER BY SOD.LIVER1
  /MISSING ANALYSIS
  /POSTHOC=DUNCAN ALPHA(0.05).
```

Oneway

Notes

Output Created		15-JUN-2021 05:38:54
Comments		
Input	Data	D:\Eman\الدكتوراه\PhD\PhD الكتابه\نتايج الاحصاء\New folder\SOD.LIVER.sav
	Active Dataset	DataSet1
	Filter	<none>
	Weight	<none>
	Split File	<none>
	N of Rows in Working Data File	24

Missing Value Handling	Definition of Missing	User-defined missing values are treated as missing.
	Cases Used	Statistics for each analysis are based on cases with no missing data for any variable in the analysis.
Syntax		ONEWAY SOD.LIVER BY SOD.LIVER1 /MISSING ANALYSIS /POSTHOC=DUNCAN ALPHA(0.05).
Resources	Processor Time	00:00:00.02
	Elapsed Time	00:00:00.06

```
[DataSet1] D:\Eman\الدكتوراه\PhD\PhD الاحصاء نتايج\الكتابه\New
folder\SOD.LIVER.sav
```

ANOVA

SOD.LIVER

	Sum of Squares	df	Mean Square	F	Sig.
Between Groups	1236.765	3	412.255	87.164	.000
Within Groups	94.593	20	4.730		
Total	1331.358	23			

Post Hoc Tests

Homogeneous Subsets

SOD.LIVER

Duncan

SOD.LIVER1	N	Subset for alpha = 0.05		
		1	2	3
2.00	6	11.0167		
3.00	6		22.6167	
1.00	6			28.5833
4.00	6			28.6167
Sig.		1.000	1.000	.979

Means for groups in homogeneous subsets are displayed.

a. Uses Harmonic Mean Sample Size = 6.000.

```
GET
  FILE='D:\Eman\_75;ه;75_;85_و;78_ك;83_ل\PhD\PhD
_75;69_;75_;89_;81_;75_ل;75_ ;80_ي;75_;78_ن\ه;76_;75_;78_لـك;\New
folder\GSH.LIVER.sav'.
DATASET NAME DataSet1 WINDOW=FRONT.
MEANS TABLES=GSH.LIVER BY GSH.LIVER1
  /CELLS MEAN SEMEAN.
```

Means

Notes

Output Created		15-JUN-2021 05:08:06
Comments		
Input	Data	D:\Eman\الدكتوراه\PhD\PhD الكتابه\نتايج الاحصاء\New folder\GSH.LIVER.sav
	Active Dataset	DataSet1
	Filter	<none>
	Weight	<none>
	Split File	<none>
	N of Rows in Working Data File	24

Missing Value Handling	Definition of Missing	For each dependent variable in a table, user-defined missing values for the dependent and all grouping variables are treated as missing.
	Cases Used	Cases used for each table have no missing values in any independent variable, and not all dependent variables have missing values.
Syntax		MEANS TABLES=GSH.LIVER BY GSH.LIVER1 /CELLS MEAN SEMEAN.
Resources	Processor Time	00:00:00.02
	Elapsed Time	00:00:00.02

```
[DataSet1] D:\Eman\الدكتوراه\PhD\PhD نتايج الاحصاء\الكتابه\New
folder\GSH.LIVER.sav
```

Case Processing Summary

	Cases					
	Included		Excluded		Total	
	N	Percent	N	Percent	N	Percent
GSH.LIVER * GSH.LIVER1	24	100.0%	0	0.0%	24	100.0%

Report

GSH.LIVER

GSH.LIVER1	Mean	Std. Error of Mean
1.00	57.2000	.93879
2.00	22.3333	.40716
3.00	46.5000	.64187

4.00	50.5333	2.10534
Total	44.1417	2.80139

```
ONEWAY GSH.LIVER BY GSH.LIVER1
  /MISSING ANALYSIS
  /POSTHOC=DUNCAN ALPHA(0.05).
```

Oneway

Notes

Output Created		15-JUN-2021 05:08:58
Comments		
Input	Data	D:\Eman\الدكتوراه\PhD\PhD الكتابه\نتايج الاحصاء\New folder\GSH.LIVER.sav
	Active Dataset	DataSet1
	Filter	<none>
	Weight	<none>
	Split File	<none>
	N of Rows in Working Data File	24
Missing Value Handling	Definition of Missing	User-defined missing values are treated as missing.
	Cases Used	Statistics for each analysis are based on cases with no missing data for any variable in the analysis.
Syntax		ONEWAY GSH.LIVER BY GSH.LIVER1 /MISSING ANALYSIS /POSTHOC=DUNCAN ALPHA(0.05).
Resources	Processor Time	00:00:00.03
	Elapsed Time	00:00:00.03

[DataSet1] D:\Eman\الدكتوراه\PhD\PhD الاحصاء نتايج\الكتابه\New folder\GSH.LIVER.sav

ANOVA

GSH.LIVER

	Sum of Squares	df	Mean Square	F	Sig.
Between Groups	4155.232	3	1385.077	156.730	.000
Within Groups	176.747	20	8.837		
Total	4331.978	23			

Post Hoc Tests

Homogeneous Subsets

GSH.LIVER

Duncan

GSH.LIVER1	N	Subset for alpha = 0.05			
		1	2	3	4
2.00	6	22.3333			
3.00	6		46.5000		
4.00	6			50.5333	
1.00	6				57.2000
Sig.		1.000	1.000	1.000	1.000

Means for groups in homogeneous subsets are displayed.

a. Uses Harmonic Mean Sample Size = 6.000.

```
GET
  FILE='D:\Eman\_75; ;80_ي;75_;78_ن\ه;75_;85_و;78_ك;83_ل
69_;75_;89_;81_;75_ل;75_;\New folder\LPO.LIVER.sav'.
DATASET NAME DataSet1 WINDOW=FRONT.
MEANS TABLES=LPO.LIVER BY LPO.LIVER1
  /CELLS MEAN SEMEAN.
```

Means

Notes

Output Created		23-FEB-2021 16:54:07
Comments		
Input	Data	D:\Eman\ الدكتوراه\نتايج الاحصاء\New folder\LPO.LIVER.sav
	Active Dataset	DataSet1
	Filter	<none>
	Weight	<none>
	Split File	<none>
	N of Rows in Working Data File	24
Missing Value Handling	Definition of Missing	For each dependent variable in a table, user-defined missing values for the dependent and all grouping variables are treated as missing.
	Cases Used	Cases used for each table have no missing values in any independent variable, and not all dependent variables have missing values.

Syntax		MEANS TABLES=LPO.LIVER BY LPO.LIVER1 /CELLS MEAN SEMEAN.
Resources	Processor Time	00:00:00.00
	Elapsed Time	00:00:00.00

```
[DataSet1] D:\Eman\الدكتوراه\نتايج الاحصاء\New folder\LPO.LIVER.sav
```

Case Processing Summary

	Cases					
	Included		Excluded		Total	
	N	Percent	N	Percent	N	Percent
LPO.LIVER * LPO.LIVER1	24	100.0%	0	0.0%	24	100.0%

Report

LPO.LIVER

LPO.LIVER1	Mean	Std. Error of Mean
1.00	16.7333	.42635
2.00	77.8333	.65557
3.00	24.4333	.27406
4.00	17.9667	.23758
Total	34.2417	5.28695

```
ONEWAY LPO.LIVER BY LPO.LIVER1
  /MISSING ANALYSIS
  /POSTHOC=DUNCAN ALPHA(0.05).
```

Oneway

Notes

Output Created		23-FEB-2021 16:54:26
Comments		
Input	Data	D:\Eman\ الدكتوراه\نتايج الاحصاء\New folder\LPO.LIVER.sav
	Active Dataset	DataSet1
	Filter	<none>
	Weight	<none>
	Split File	<none>
	N of Rows in Working Data File	24
Missing Value Handling	Definition of Missing	User-defined missing values are treated as missing.
	Cases Used	Statistics for each analysis are based on cases with no missing data for any variable in the analysis.
Syntax		ONEWAY LPO.LIVER BY LPO.LIVER1 /MISSING ANALYSIS /POSTHOC=DUNCAN ALPHA(0.05).
Resources	Processor Time	00:00:00.03
	Elapsed Time	00:00:00.03

```
[DataSet1] D:\Eman\الدكتوراه\نتايج الاحصاء\New folder\LPO.LIVER.sav
```

ANOVA

LPO.LIVER

	Sum of Squares	df	Mean Square	F	Sig.
Between Groups	15407.125	3	5135.708	4607.394	.000
Within Groups	22.293	20	1.115		
Total	15429.418	23			

Post Hoc Tests

Homogeneous Subsets

LPO.LIVER

Duncan

LPO.LIVER1	N	Subset for alpha = 0.05		
		1	2	3
1.00	6	16.7333		
4.00	6	17.9667		
3.00	6		24.4333	
2.00	6			77.8333
Sig.		.057	1.000	1.000

Means for groups in homogeneous subsets are displayed.

a. Uses Harmonic Mean Sample Size = 6.000.

```
GET
  FILE='D:\69_;75_;89_;81_;75_ل;75_ ;80_ي;75_;78_ن;\New folder\GPX.LIVER..sav'.
DATASET NAME DataSet1 WINDOW=FRONT.
MEANS TABLES=GPX.LIVER BY GPX.LIVER1
  /CELLS MEAN SEMEAN.
```

Means

Notes

Output Created	20-FEB-2021 07:03:13
Comments	

Input	Data	D:\نتايج الاحصاء\New folder\GPX.LIVER..sav
	Active Dataset	DataSet1
	Filter	<none>
	Weight	<none>
	Split File	<none>
	N of Rows in Working Data File	24
Missing Value Handling	Definition of Missing	For each dependent variable in a table, user-defined missing values for the dependent and all grouping variables are treated as missing.
	Cases Used	Cases used for each table have no missing values in any independent variable, and not all dependent variables have missing values.
Syntax		MEANS TABLES=GPX.LIVER BY GPX.LIVER1 /CELLS MEAN SEMEAN.
Resources	Processor Time	00:00:00.02
	Elapsed Time	00:00:00.02

[DataSet1] D:\نتايج الاحصاء\New folder\GPX.LIVER..sav

Case Processing Summary

	Cases					
	Included		Excluded		Total	
	N	Percent	N	Percent	N	Percent
GPX.LIVER * GPX.LIVER1	24	100.0%	0	0.0%	24	100.0%

Report

GPX.LIVER

GPX.LIVER1	Mean	Std. Error of Mean
1.00	116.7667	.75748
2.00	53.5667	.49777
3.00	106.0833	.97789
4.00	116.1667	.36301
Total	98.1458	5.44871

```
ONEWAY GPX.LIVER BY GPX.LIVER1
  /MISSING ANALYSIS
  /POSTHOC=DUNCAN ALPHA(0.05).
```

Oneway

Notes

Output Created		20-FEB-2021 07:03:42
Comments		
Input	Data	D:\نتايج الاحصاء\New folder\GPX.LIVER..sav
	Active Dataset	DataSet1
	Filter	<none>
	Weight	<none>
	Split File	<none>
	N of Rows in Working Data File	24
Missing Value Handling	Definition of Missing	User-defined missing values are treated as missing.
	Cases Used	Statistics for each analysis are based on cases with no missing data for any variable in the analysis.

Syntax		ONEWAY GPX.LIVER BY GPX.LIVER1 /MISSING ANALYSIS /POSTHOC=DUNCAN ALPHA(0.05).
Resources	Processor Time	00:00:00.03
	Elapsed Time	00:00:00.03

[DataSet1] D:\الاحصاء نتايج\New folder\GPX.LIVER..sav

ANOVA

GPX.LIVER

	Sum of Squares	df	Mean Square	F	Sig.
Between Groups	16330.751	3	5443.584	1900.416	.000
Within Groups	57.288	20	2.864		
Total	16388.040	23			

Post Hoc Tests

Homogeneous Subsets

GPX.LIVER

Duncan

GPX.LIVER1	N	Subset for alpha = 0.05		
		1	2	3
2.00	6	53.5667		
3.00	6		106.0833	
4.00	6			116.1667

1.00	6			116.7667
Sig.		1.000	1.000	.546

Means for groups in homogeneous subsets are displayed.

a. Uses Harmonic Mean Sample Size = 6.000.

Means

Notes

Output Created		12-NOV-2021 01:37:17
Comments		
Input	Active Dataset	DataSet0
	Filter	<none>
	Weight	<none>
	Split File	<none>
	N of Rows in Working Data File	24
Missing Value Handling	Definition of Missing	For each dependent variable in a table, user-defined missing values for the dependent and all grouping variables are treated as missing.
	Cases Used	Cases used for each table have no missing values in any independent variable, and not all dependent variables have missing values.
Syntax		MEANS TABLES=KI67 BY KI671 /CELLS MEAN SEMEAN.
Resources	Processor Time	00:00:00.00
	Elapsed Time	00:00:00.01

[DataSet0]

Case Processing Summary

	Cases					
	Included		Excluded		Total	
	N	Percent	N	Percent	N	Percent
KI67 * KI671	24	100.0%	0	0.0%	24	100.0%

Report

KI67

KI671	Mean	Std. Error of Mean
1.00	1.0163	.04612
2.00	3.1104	.06741
3.00	1.6965	.04314
4.00	1.2552	.02578
Total	1.7696	.17071

```
ONEWAY KI67 BY KI671
  /MISSING ANALYSIS
  /POSTHOC=DUNCAN ALPHA(0.05).
```

Oneway

Notes

Output Created		12-NOV-2021 01:39:53
Comments		
Input	Active Dataset	DataSet0
	Filter	<none>
	Weight	<none>
	Split File	<none>

	N of Rows in Working Data File	24
Missing Value Handling	Definition of Missing	User-defined missing values are treated as missing.
	Cases Used	Statistics for each analysis are based on cases with no missing data for any variable in the analysis.
Syntax		ONEWAY KI67 BY KI671 /MISSING ANALYSIS /POSTHOC=DUNCAN ALPHA(0.05).
Resources	Processor Time	00:00:00.03
	Elapsed Time	00:00:00.08

[DataSet0]

ANOVA

KI67

	Sum of Squares	df	Mean Square	F	Sig.
Between Groups	15.811	3	5.270	382.040	.000
Within Groups	.276	20	.014		
Total	16.086	23			

Post Hoc Tests

Homogeneous Subsets

KI67

Duncan

KI671	N	Subset for alpha = 0.05			
		1	2	3	4
1.00	6	1.0163			
4.00	6		1.2552		
3.00	6			1.6965	
2.00	6				3.1104
Sig.		1.000	1.000	1.000	1.000

Means for groups in homogeneous subsets are displayed.
