# Supplementary figures and images for: Protective Effects of Naringin–Dextrin Nanoformula against Chemically Induced Hepatocellular Carcinoma in Wistar Rats: Roles of Oxidative Stress, Inflammation, Cell Apoptosis, and Proliferation
Source: Pharmaceuticals (Basel). 2022 Dec 14;15(12):1558. doi: 10.3390/ph15121558 (PMC9786090; doi:10.3390/ph15121558)

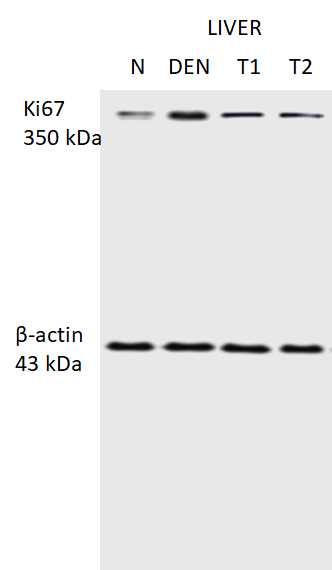

Supplement: Supplementary file 1 [file pharmaceuticals-15-01558-s001.zip › Supplementary file/Ki-67 one replicate.jpg]

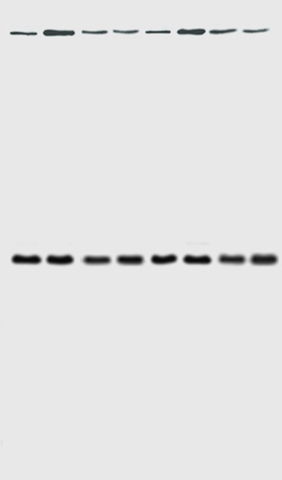

Supplement: Supplementary file 1 [file pharmaceuticals-15-01558-s001.zip › Supplementary file/Ki-67 two replicates.jpg]
